# Ligand regulation and function of preformed EGFR dimers

**DOI:** 10.1073/pnas.2602436123

**Published:** 2026-07-20

**Authors:** Yuhong Zuo, Hillel T. Schwartz, Kahlil Walker, Long Han, Paul W. Sternberg, Kathryn M. Ferguson

**Affiliations:** ^a^ Department of Pharmacology, Yale University School of Medicine, New Haven, CT 06520; ^b^ Cancer Biology Institute, Yale University, West Haven, CT 06516; ^c^ Division of Biology and Biological Engineering, California Institute of Technology, Pasadena, CA 91125

**Keywords:** receptor tyrosine kinases, EGFR, preformed dimers, growth factor signaling, cryo-EM

## Abstract

Receptor tyrosine kinases (RTKs) are key therapeutic targets in cancer, diabetes, and other diseases. With only one transmembrane α-helix—compared with seven in G-protein-coupled receptors—RTKs are thought to be activated by ligand-induced dimerization. Complicating this view, however, one of the best-studied RTKs, the insulin receptor (IR), forms allosterically regulated covalent dimers. Moreover, noncovalent “preformed” dimers have frequently been reported for the sequence-related epidermal growth factor receptor (EGFR), one of the first RTKs for which ligand-induced dimerization was described. Here, we describe a detailed structural view of a preformed EGFR dimer. Using cryo-EM, we describe how the *Caenorhabditis elegans* EGFR (LET-23) dimerizes without ligand. We show that preformed dimer formation modulates ligand sensitivity in vivo, but is not required for signaling itself. We also elucidate substantial ligand-induced conformational changes in LET-23 required for signaling. Our structures reveal unexpected similarities between regulation of LET-23 and the IR, suggesting that LET-23 may represent an evolutionary “missing link” between the IR and EGFR families. In the absence of ligand, intermolecular interactions within preformed receptor dimers hold the extracellular juxtamembrane regions far apart to separate the intracellular kinase domains so that they remain inactive. Ligand binding disrupts these interactions to remove the restraints on the kinase domains, which then can associate to become activated. Our analysis further suggests a unified model for the allosteric activation of preformed RTK dimers that has important implications for understanding cell-surface EGFR.

## Significance

The epidermal growth factor receptor (EGFR) is generally considered to be activated by ligand-induced dimerization. Confounding this view, however, is significant evidence for unliganded dimers of EGFR at the cell surface. This study provides a detailed structural view of a preformed EGFR dimer—a structurally undercharacterized state of EGFR—specifically involving *Caenorhabditis elegans* EGFR (LET-23). Ligand-induced changes in this preformed dimer suggest an activation mechanism that is remarkably similar to the allosteric activation of the covalent insulin receptor dimer. This mechanistic connection between these two important receptor tyrosine kinase families advances our understanding of EGFR regulation at the cell surface and has significant implications for the development and application of targeted therapies.

The epidermal growth factor receptor (EGFR) and insulin receptor (IR) families are among the most extensively studied receptor tyrosine kinases (RTKs), in part due to their important roles in many diseases including cancer and diabetes (1–4). Despite sharing a structurally and functionally similar ligand-binding module that is only seen in these two RTK families (5, 6), the mechanisms of activation of the EGFR and IR families have generally been considered to be distinct. IR family receptors form disulfide-linked dimers that are activated by ligand-induced conformational changes (7, 8). By contrast, EGFR is thought to be activated by ligand-induced receptor dimerization (9), which is also considered to be a component of the activation mechanism for all RTKs; the IR family being the exception (6). Although EGFR was one of the first RTKs for which ligand-induced dimerization was described (9), the distinction between dimerizing and nondimerizing RTKs has since been blurred by the many cellular studies that describe “preformed” dimers of EGFR at the cell in the absence of ligand (10–17), although their role is controversial and no structure has been described.

Here we use the EGFR of *Caenorhabditis elegans*, LET-23 (18), to gain insight into the structure and mechanistic significance of preformed EGFR dimers. The purified extracellular region (ECR) of LET-23 (sLET-23) was previously shown to dimerize strongly even in the absence of ligand (19), contrasting with the ECRs from human EGFR family members, which form only monomers (20–22). Taking advantage of this observation, we used single-particle cryogenic electron microscopy (cryo-EM) to determine the structure of the preformed LET-23 ECR. Intermolecular interactions within this preformed receptor dimer hold the extracellular juxtamembrane regions far apart to separate the intracellular kinase domains so that they remain inactive. We show that disrupting interactions that stabilize the preformed dimer sensitizes LET-23 to activation in vivo, but that the preformed dimers are not required for receptor signaling. We further show that ligand binding induces major conformational changes that remove restraints on the kinase domains, allowing them to associate and become activated. The mechanism of activation of this preformed EGFR dimer suggested by our studies closely resembles mechanisms proposed for IR family of RTKs. Thus, our structural and in vivo data provide a generalized mechanism of RTK activation while suggesting how preformed dimers can influence regulation of receptor activity.

## Results and Discussion

### Structure of a Preformed EGFR Dimer.

**Fig. 1.**
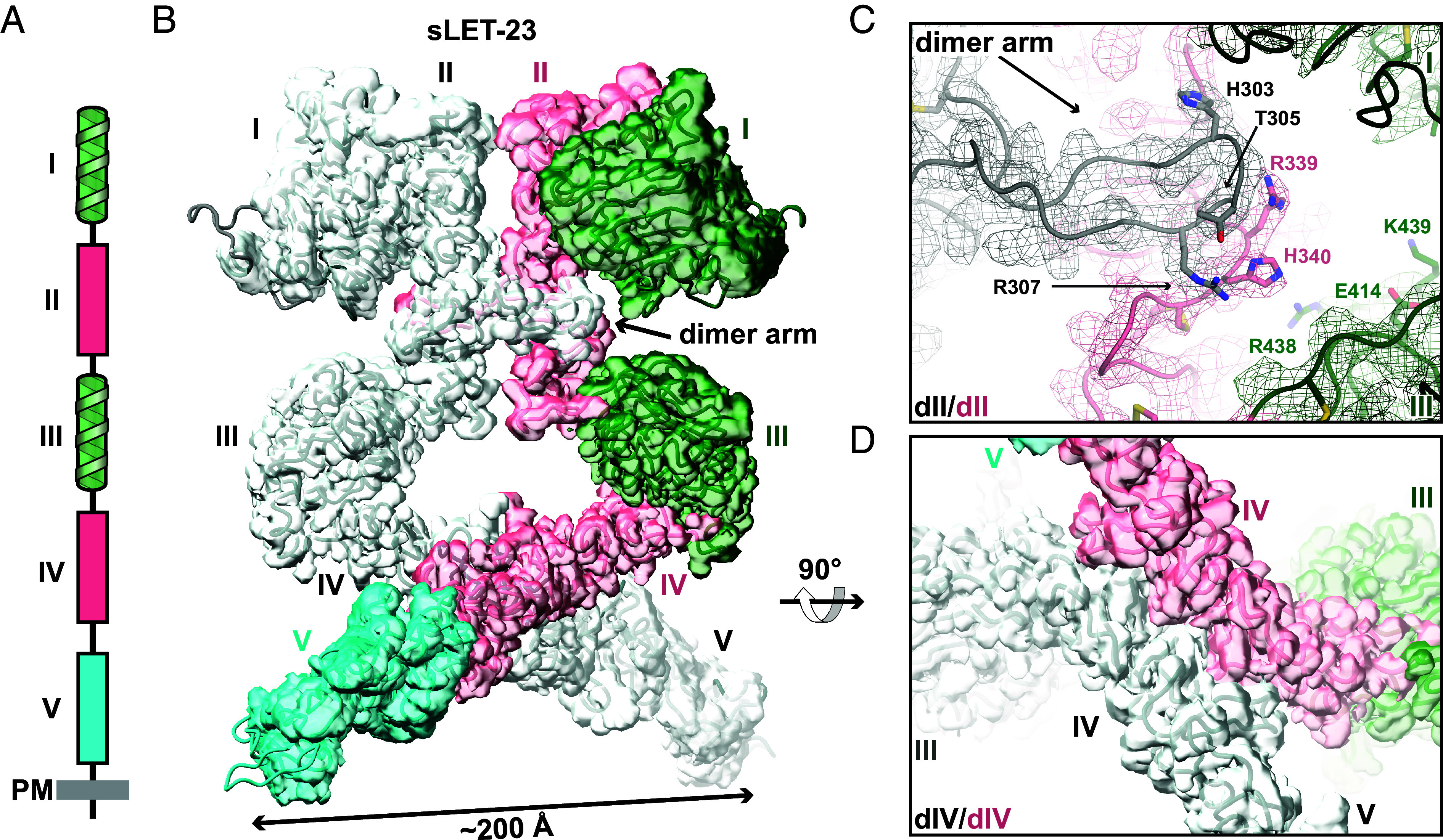
Structure of a preformed unliganded EGFR dimer. (*A*) The domain architecture of LET-23 ECR is shown with domains I and III in green, cysteine-rich domains II and IV in salmon, and V in cyan. The horizontal gray bar indicates the position of the plasma membrane (PM). (*B*) Cryo-EM map and structural model for the unliganded sLET-23 dimer. For one protomer domains are colored as in *A*. The other protomer is white. The 200 Å separation between membrane proximal ends of the two protomers is indicated. (*C*) A view of the domain II (dII) interface showing the well-ordered dimer arm. Colors are as in *A*, key side chains shown in stick representation and the cryo-EM density is in mesh representation colored by domain. (*D*) A representation as in *A*, viewed from below—a 90° counterclockwise rotation about the x-axis, to show the domain IV (dIV) interface.

We obtained three-dimensional reconstructions of the complete LET-23 ECR (domains I–V; Fig. 1*A* and *SI Appendix*, Figs. S1*A* and S2) with overall resolution of 2.9 Å. As shown in Fig. 1*B*, much of the dimer interface (58%: 2,000 Å^2^ buried) is contributed by the cysteine-rich domain II (dII), centered on a well resolved “dimer arm” (Fig. 1 *B* and *C*) that plays a key role in dimerization of the ligand-bound human EGFR (23–25) (see below). An additional unique contribution to the sLET-23 dimer interface is made by the second cysteine-rich domain (domain IV, dIV) through a set of interactions that contribute the remaining 42% of the dimer interface (1,430 Å^2^ buried; Fig. 1*D*).

These domain IV/domain IV (dIV/dIV) interactions reveal an unanticipated role for domain V (dV), which is seen only in invertebrate EGFRs. Domain V is very similar in structure to dIV, and effectively extends it so that domains IV and V together form a 140 Å-long rigid rod or “leg.” The dIV/dIV interactions constrain these legs in sLET-23 so that they are separated by some 200 Å as they would enter the plasma membrane. This conformation would keep the intracellular kinase domain-containing regions of the receptor well separated to prevent receptor activation, in a manner similar to that suggested for autoinhibition of the insulin receptor (IR) (26).

### LET-23 Activation by Ligand.

**Fig. 2.**
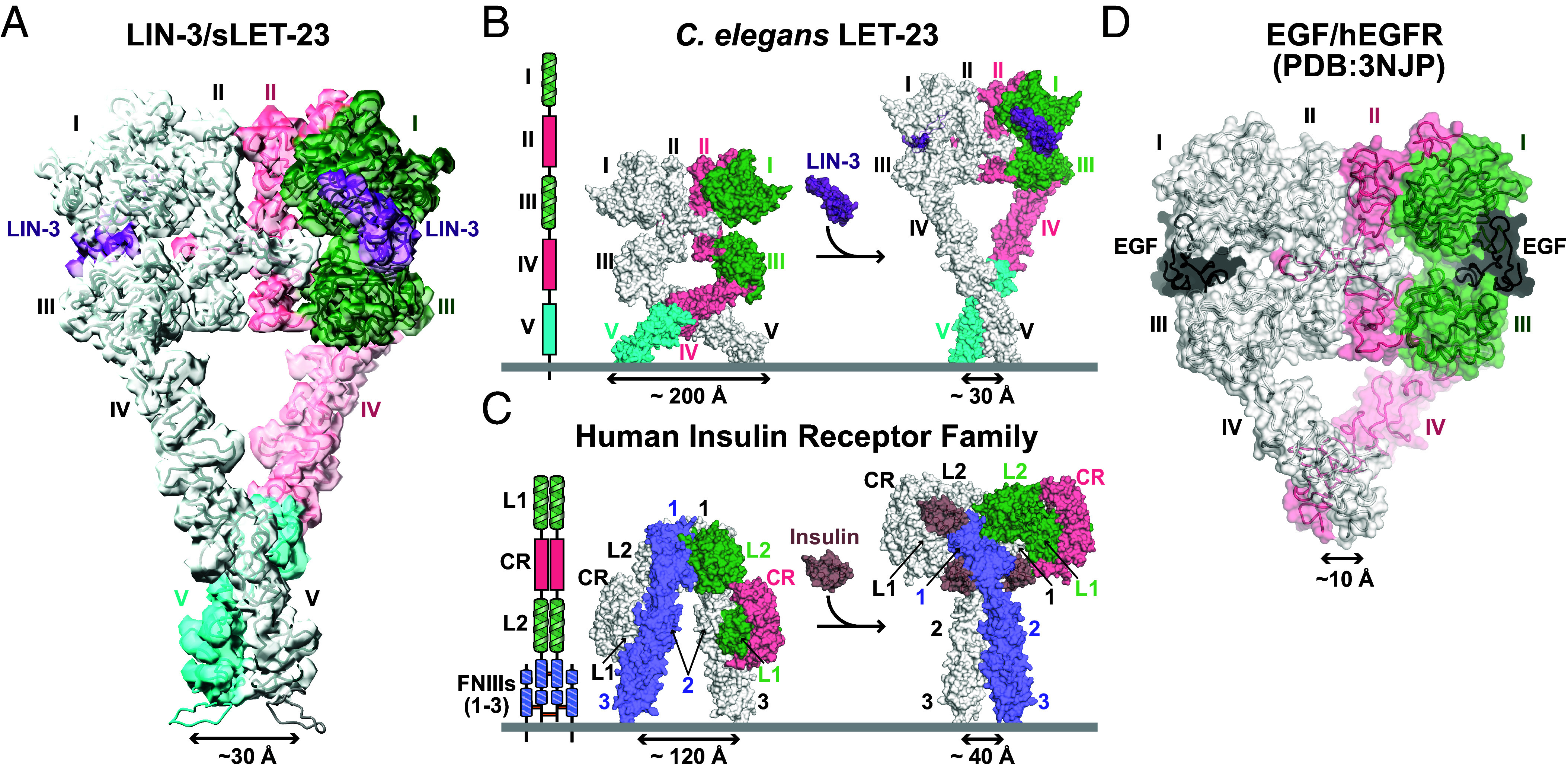
Structures of the LET-23 ECR suggest an IR-like activation mechanism. (*A*) Cryo-EM map and structural model for a LIN-3/sLET-23 dimer, colored as in Fig. 1*B* with LIN-3 in purple. The ~30 Å separation between membrane proximal ends of the two protomers is indicated. (*B*) Ligand-induced conformational changes in LET-23. The domain architecture of sLET-23 (*Left*) and surface representations of the unliganded (*Middle*) and ligand-bound (*Right*) LET-23 are shown colored as in *A*. Upon ligand-binding, the membrane-proximal ends of the ECR are brought together. The horizontal gray bar indicates the position of the plasma membrane. (*C*) Ligand-induced changes in IR. The domain architecture of IR (*Left*) and surface representations of unliganded (*Middle*) and ligand-bound (*Right*) IR are shown. Domains L1–CR–L2 are equivalent to domains I–II–III in EGFR (5) and are colored as in *B*. The IR FNIII domains are in blue. Interchain disulfide bonds are indicated with orange bars. Ligand-binding induces a major conformational change that brings the membrane proximal ends of the ECR from 120 Å separation in unliganded IR (PDB:4ZXB) (29) to 40 Å when ligand binds—here represented as the symmetric complex formed at saturating ligand concentrations (PDB:6PXV) (31). (*D*) The EGF bound human EGFR (hEGFR) dimer (PDB:3NJP) (32) in ribbon view with a transparent molecular surface, colored as in *A* with EGF in black.

We next used cryo-EM to ask how the sLET-23 dimer is altered by binding of the activating ligand, LIN-3 (27). We could refine the most abundant conformation of the LIN-3/sLET-23 complex to 2.37 Å resolution (*SI Appendix*, Fig. S3), which revealed a dramatically different conformation from that seen for unliganded sLET-23 (Fig. 2 *A* and *B*). The most striking change is that the C-termini of the two (membrane-proximal) copies of dV in the dimer are brought to within 30 Å of one another rather than being separated by ~200 Å as they are in the unliganded dimer (Fig. 2*B*). This transition is highly reminiscent of that seen in the IR dimer upon ligand binding (Fig. 2*C*) (8, 26). The three FNIII domains in the IR ECR form legs of similar length to those formed by domains IV and V of LET-23 and are held apart by intermolecular interactions in the absence of ligand—so that the TM domains are predicted to be separated by ~120 Å (26, 28, 29). Binding of insulin to the L1 and L2 domains of IR (equivalent to domains I and III in LET-23) breaks intramolecular interactions and allows the legs to come together (30, 31), as they do in ligand-bound LET-23. Thus, both the unliganded LET-23 and IR dimers have strong autoinhibitory intermolecular interactions that serve to keep the intracellular kinase domain-containing regions apart until they are “relieved” by ligand binding to allow receptor activation.

**Fig. 3.**
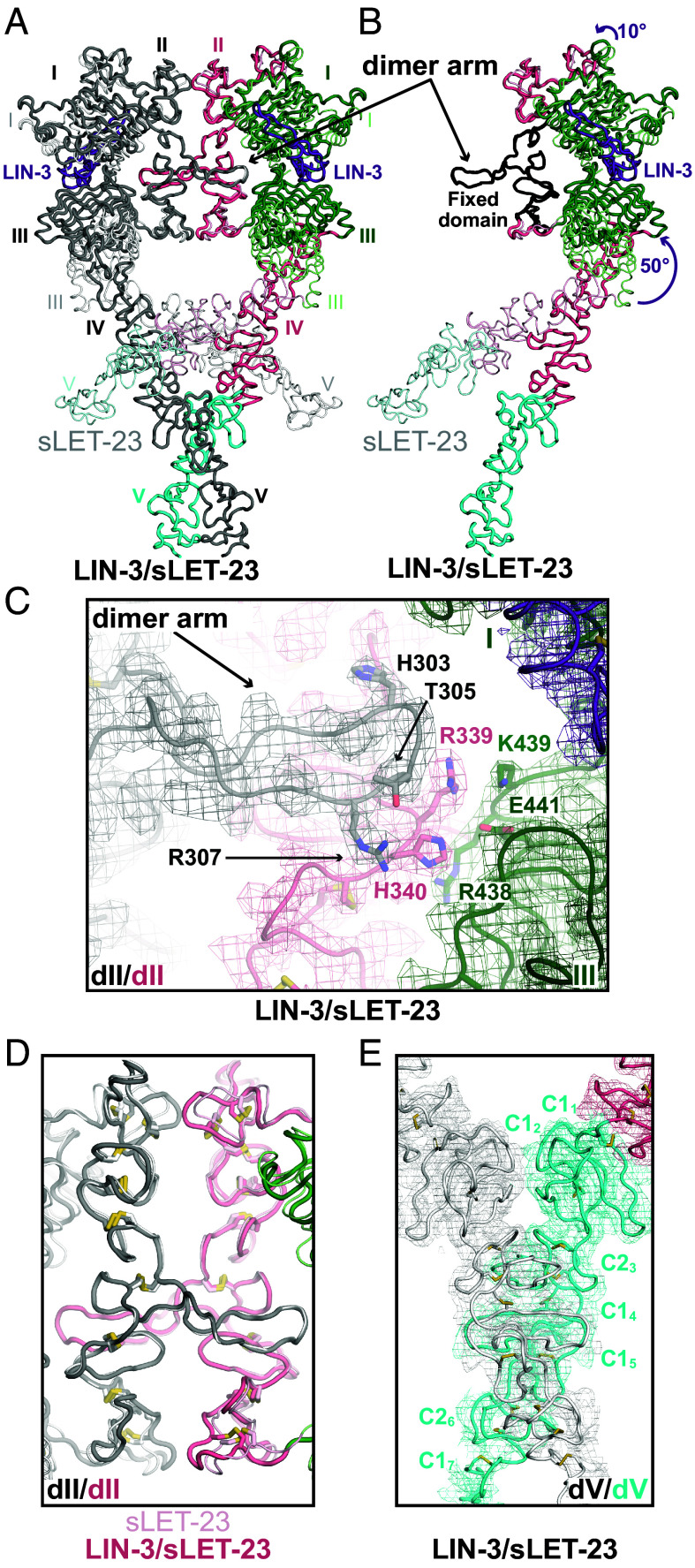
Ligand-induced conformational changes in sLET-23. (*A*) The unliganded and ligand-bound dimers of sLET-23 are shown in ribbon representation, superimposed using the dimer arms [amino acids (aa) 295–322]. Domains are colored as in Fig. 2*A*, with the unliganded structure in a thinner, lighter-colored ribbon. (*B*) The right-hand protomers in *A* are shown highlighting the conformational changes. The transformation from unliganded sLET-23 to ligand-bound LIN-3/sLET-23 can be described as: *i*) An ~50° rotation of the domain III–IV–V module about an axis approximately parallel to the dimer arm, and *ii*) An ~10° rotation of dI and the first three disulfide-bonded modules of dII about an axis perpendicular to the dimer arm. (*C*) A view of the dII dimerization interface orientated as Fig. 1*C*. Domain III (green) packs against dII (salmon) close to the dimer arm of the opposite protomer (white). Side chains shown are the same as in Fig. 1*C*. The cryo-EM density is shown in mesh representation colored by domain. (*D*) A closeup view of the dII dimer interface of the unliganded and ligand-bond dimers, colored as in *A*. The buttressing of dII by domains I and III induces a bend in dII, bringing the two ends of this part of dII closer together. (*E*) A ribbon representation of the LIN-3/sLET-23 dV region. The cryo-EM density for a dV locally refined map is shown in mesh representation, colored as for the ribbon. Disulfide-bonded modules are marked. View is an ~90° rotation about the y-axis relative to *A*.

The dramatic overall LET-23 conformational changes shown in Fig. 2*B* arise from relatively small local changes induced when LIN-3 occupies the ligand-binding LET-23 “head,” which closely resembles that of human EGFR (Fig. 2*D*). As with EGF/EGFR, LIN-3 interacts bivalently with both domains I and III of each LET-23 protomer. In contrast to the human case, where a major (~130˚) reorientation is induced to expose the dimer arm (22, 33), LIN-3 binding to LET-23 reorients domains I and III by approximately just 10˚ and 50˚ respectively (Fig. 3 *A* and *B*) compared to the unliganded dimer. These reorientations have two consequences. First, the reoriented dIII contacts dII near the dimer arm (Fig. 3*C*), likely stabilizing the dimer. Second, the movements of both domains with respect to dII appear to “buttress” the dII dimer interface (Fig. 3*D*), making it more intimate with additional polar interactions, particularly in the upper part of the interface in Fig. 2*A*. As a result, LIN-3 binding increases the surface area buried at the dII interface by ~30%, to 2,592 Å^2^.

Importantly, all of these ligand-dependent changes in sLET-23’s conformation are accompanied by a complete loss of the dIV/dIV interactions shown in Fig. 1*D*—with dIV no longer contributing at all to the dimer interface in ligand-bound sLET-23 (Fig. 2 *A* and *B*). A different set of interactions that involve dV (Fig. 3*E*) may instead contribute to stability of the ligand-bound dimer, burying a similar area to dIV/dIV in the unliganded structure (~1,800 Å^2^), although this region is less well resolved than the rest of the structure. Intriguingly, whereas the domain IV–V legs of the unliganded LET-23 dimer cross in a well-defined right-handed sense (Figs. 1*B* and 2*B*), this is switched to a more loosely held left-handed crossing in the LIN-3-bound dimer (Fig. 2 *A* and *B*).

### LIN-3 Stabilizes Mutated sLET-23 Dimers.

**Fig. 4.**
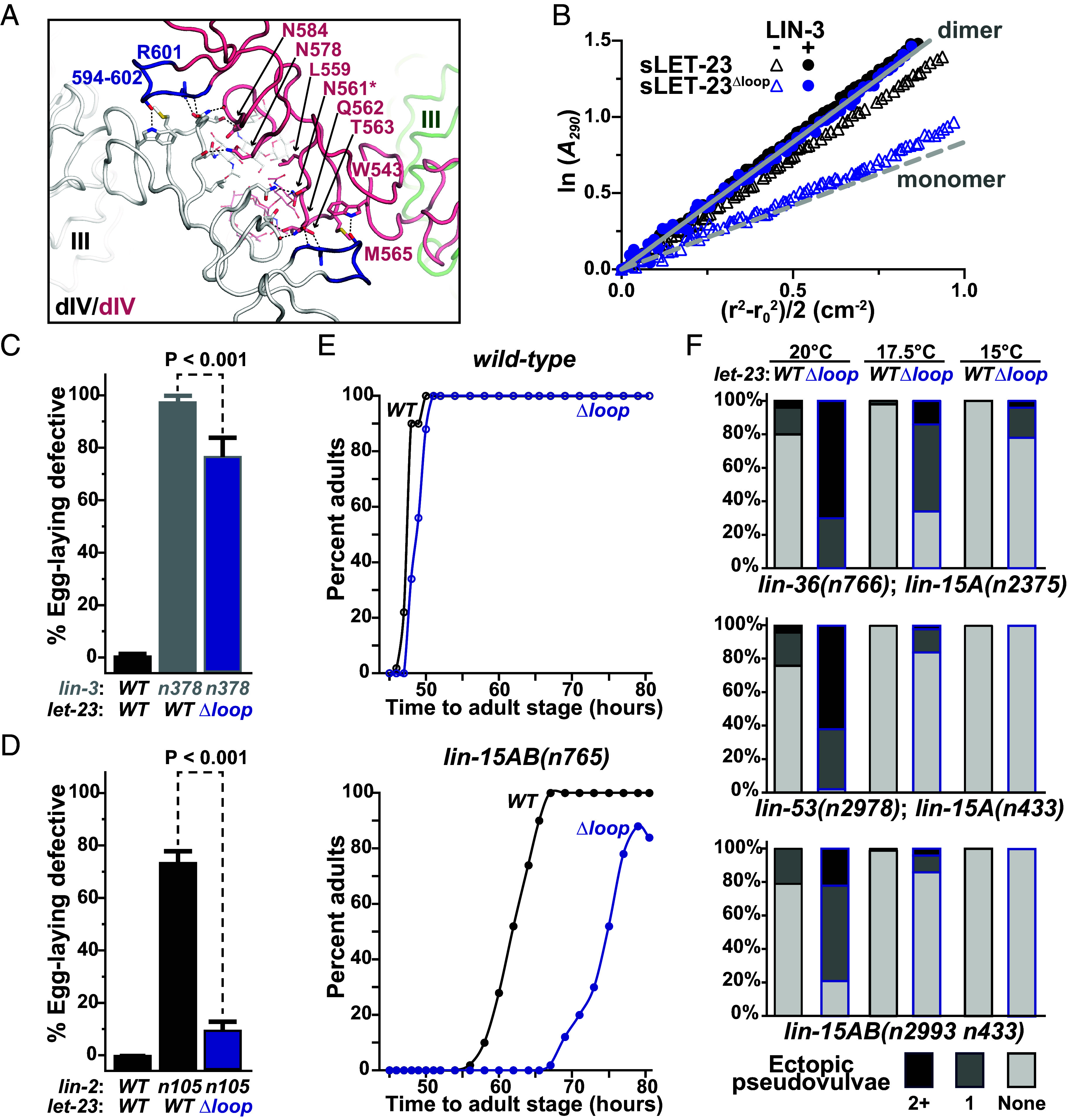
Weakening dIV interactions cause increased *let-23* pathway activity in vivo. (*A*) A ribbon representation of the dIV dimer interface, orientated and colored as in Fig. 1*D*. Side chains that make presumed hydrogen bonding interactions (black dashed lines) are shown in stick representation and labeled for the colored protomer of this symmetric interface. The dimer loop (aa 594–602) is in dark blue. Ordered N-linked glycans on each N561 (marked with an asterisk) that contribute ~200 Å^2^ of buried surface to the interface, are in ball-and-sticks representation. (*B*) SE-AUC analysis of sLET-23 and sLET-23^Δloop^ with and without 1.2-fold molar excess of LIN-3^EGF^. Representative curves for 10 μM receptor at 8,000 rpm are plotted as the natural logarithm of absorbance, ln (*A_290_*) at radial distance r plotted against (r^2^-r_0_^2^)/2. For a single species, this transformation yields a straight line with slope proportional to the molecular weight. Dashed and solid lines show expected slopes for monomer (100 kDa) and dimer (200 kDa) respectively. Data points are black for sLET-23 [taken from a study by Freed et al. (19)] and dark blue for sLET-23^Δloop^, with open triangles for the protein alone and filled circles plus LIN-3. Data are representative of at least three independent experiments (Dataset S1). (*C* and *D*) Weakening dIV interactions suppresses the Vul phenotype caused by (*C*), *lin-3*(*n378*) and (*D*) *lin-2*(*n105*), as determined by inability to lay eggs. WT indicates the wild type *let-23* genotype, and *Δloop* indicates the dIV loop deletion mutation. Values are the mean from at least three experiments, each with n = 50. Error bars are standard deviation. *P*-value determined by Fisher’s Exact Test (Datasets S2 and S3). (*E*) Effects of the *let-23* (*Δloop*) mutation on the time to develop to adulthood in a wild type (*Upper* panel, open circles) and *lin-15AB* (*n765*) (*Lower* panel, closed circles) background. The percentage of animals that reached adulthood for different elapsed times following release from developmental arrest is shown (*SI Appendix*). Each time point is a sample of 50 animals from a larger population. Whereas the *let-23Δloop* mutation has no effect in the WT background (*Upper* panel, open blue points), in the *lin-15AB* (*n765*) background onset of adulthood is delayed by an additional 25% (closed blue points) relative to *lin-15AB* (*n765*) alone (black points) (Dataset S4). (*F*) Weakening dIV interactions enhance penetrance of the weakly penetrant and expressive multivulval (Muv) phenotypes in the indicated ts synMuv mutants that have increased *lin-3 EGF* expression. Percentage of animals with no ectopic pseudovulvae (light gray), one ectopic pseudovulva (dark gray), or two or more ectopic pseudovulvae (black) are shown for growth at 20 °C, 17.5 °C, or 15 °C; n = 50 (Dataset S5).

Whereas dII plays an important role in dimerization of both unliganded and ligand-bound LIN-3, it cooperates with dIV in the absence of ligand (Fig. 1*B*) and with dV in the ligand-bound dimer (Fig. 2*A*). Indeed, dimers of unliganded sLET-23 can be disrupted by deleting dIV or by mutations in the dII dimer arm (19). Moreover, when both domains IV and V are deleted, sLET-23 shows similar ligand-induced dimerization properties to the extracellular regions of its human and *Drosophila melanogaster* orthologs (19, 20, 34). The dIV contacts seen in our structure of the unliganded sLET-23 dimer (Fig. 1*D*) are dominated by a “dIV dimer loop” that spans residues 594–602 (Fig. 4*A*) and is not conserved in the mammalian or insect EGFRs (*SI Appendix*, Fig. S1*A*). Importantly, when we deleted just this loop, we find that the resulting sLET-23^Δloop^ variant is largely monomeric at 10 μM in analytical ultracentrifugation experiments (Fig. 4*B*)—but dimerizes robustly upon LIN-3 binding.

### Role of Preformed LET-23 Dimers In Vivo.

Since removing the dIV dimer loop allows sLET-23 to behave as a monomer and display ligand-induced dimerization, we wanted to ask how such a change—substantially weakening preformed dimers—would impact LET-23 signaling in vivo. LET-23 plays a well characterized role in *C. elegans* vulval development (35). Vulval cell fate determination in *C. elegans* occurs in the third larval stage (L3), when LIN-3 secreted from the anchor cell (AC) acts on the six LET-23-expressing vulval precursor cells (VPCs) to specify an invariant pattern of vulval cell fates and promote formation of a single vulva. Increased LET-23 activation, as a result of point mutations or overexpression of LIN-3 (36–38), can promote aberrant VPC specification and lead to ectopic vulval induction (multivulva or Muv phenotype). Reduced LET-23 activation results in the vulvaless (Vul) phenotype (39, 40).

We used CRISPR-Cas9 and homology-directed repair to introduce mutations into the endogenous *let-23* locus to delete the LET-23 dIV dimer loop shown in Fig. 4*A*. Animals homozygous for the *let-23* (*Δloop*) mutation in an otherwise WT background were viable and healthy, and appeared grossly WT. They did not exhibit ectopic vulval induction. Nor did they display phenotypes associated with loss of LET-23 activity (paralysis and early larval lethality or the Vul phenotype) (39, 40). These results suggest that LET-23 signaling remains intact after deletion of the dIV dimer loop, consistent with the fact that LIN-3 can still induce dimerization of the mutated LET-23 ECR (Fig. 4*B*). Indeed, the signaling network that controls vulval development is known to be robust (41); tolerating relatively large changes in *lin-3* expression, for example (42). Our findings suggest that LET-23 signaling is also robust to reduced strength of preformed receptor dimers.

### Preformed Dimers Tune Signal Sensitivity.

With no apparent effect of LET-23 dIV mutations in a WT *C. elegans* background, we next asked whether using distinct partial loss-of-function alleles in the *lin-3-let-23* signaling axis might increase sensitivity to mutations that weaken preformed LET-23 dimers. We used partially penetrant mutations in the upstream *let-23* regulators *lin-3 EGF* (which encodes LET-23’s ligand) (27) and *lin-2 CASK* (required for correct LET-23 location in the VPC basolateral membranes) (43–45). For the *lin-3* (*n378*) mutant, 98 ± 2% of homozygotes lacked a functional vulva and were unable to lay eggs (46) (Fig. 4*C*), and the *let-23* (*Δloop*) mutation significantly suppressed this egg-laying defect (to 77 ± 7% Vul). In the less penetrant *lin-2* (*n105*) background (44), 73 ± 4% of homozygotes failed to lay eggs, and the *let-23* (*Δloop*) mutation almost completely suppressed this phenotype, such that fewer than 10% of the doubly mutated animals exhibited the egg-laying defect (Fig. 4*D*). These data indicate that disrupting dIV interactions in the preformed LET-23 dimer sensitizes the receptor to ligand-induced activation. We also tested the effects of less extensive dIV dimer interface mutations, mutating L559, Q562, and M565 to alanine in *let-23* (*3A*), or L559, N561, Q562, and M565 to alanine in *let-23* (*4A*) (*SI Appendix*, Fig. S4*A*). Neither *let-23* (*3A*) nor *let-23* (*4A*) altered the *lin-3* (*n378*) Vul phenotype (*SI Appendix*, Fig. S4*B*), but had modest effects on the *lin-2* (*n105*) Vul phenotype (*SI Appendix*, Fig. S4*C*).

Just as mutations in the LET-23 dIV dimer loop reduce penetrance of partial loss of function alleles in the *lin-3-let-23* signaling axis, we also found that they *increase* penetrance of mutations that increase *let-23* signaling in synthetic multivulva (synMuv) animals (47–49). The *lin-15AB* (*n765*) synMuv mutant has temperature sensitive (ts) mutations in the *lin-15A* and *lin-15B* genes that increase *lin-3* expression to cause the Muv phenotype and delay development to adulthood at 20 °C (Fig. 4*E*) (39, 50). Introducing the *let-23* (*Δloop*) mutation in the *lin-15AB* (*n765*) background strongly enhanced the slow larval development phenotype of that mutant (Fig. 4 *E*, *Lower*). The penetrance of the multivulval phenotype at 15 °C was also increased from 79% for *lin-15* (*n765*) alone to 95% for the *let-23* (*Δloop*); *lin-15* (*n765*) double mutant (n = 100), with larger pseudovulvae in the double relative to the single mutant. We also introduced the *let-23* (*Δloop*) mutation into three other weakly penetrant synMuv double mutants (47, 49): *i*) *lin-36* (*n766*); *lin-15A* (*n2375*), *ii*) *lin-53* (*n2978*); *lin-15A* (*n433*), and *iii*) *lin-15AB* (*n2993 n433*), and found that it substantially increased penetrance in all cases (Fig. 4*F*). The *let-23* (*3A*) and *let-23* (*4A*) alanine substitution mutations also sensitized LET-23 to LIN-3 in these contexts, but to a lesser extent (*SI Appendix*, Fig. S4 *D* and *E*).

Together these data show that weakening the ability of dIV to stabilize the preformed LET-23 dimer increases the sensitivity of the receptor to LIN-3. Thus, the preformed dimer does not seem to be required for LET-23 signaling per se, but appears to play a key role in defining the sensitivity of the *lin-3-let-23* signaling axis to changes; tuning this signaling axis and defining its robustness and likely variability.

### Cryo-EM of sLET-23^Δloop^.

**Fig. 5.**
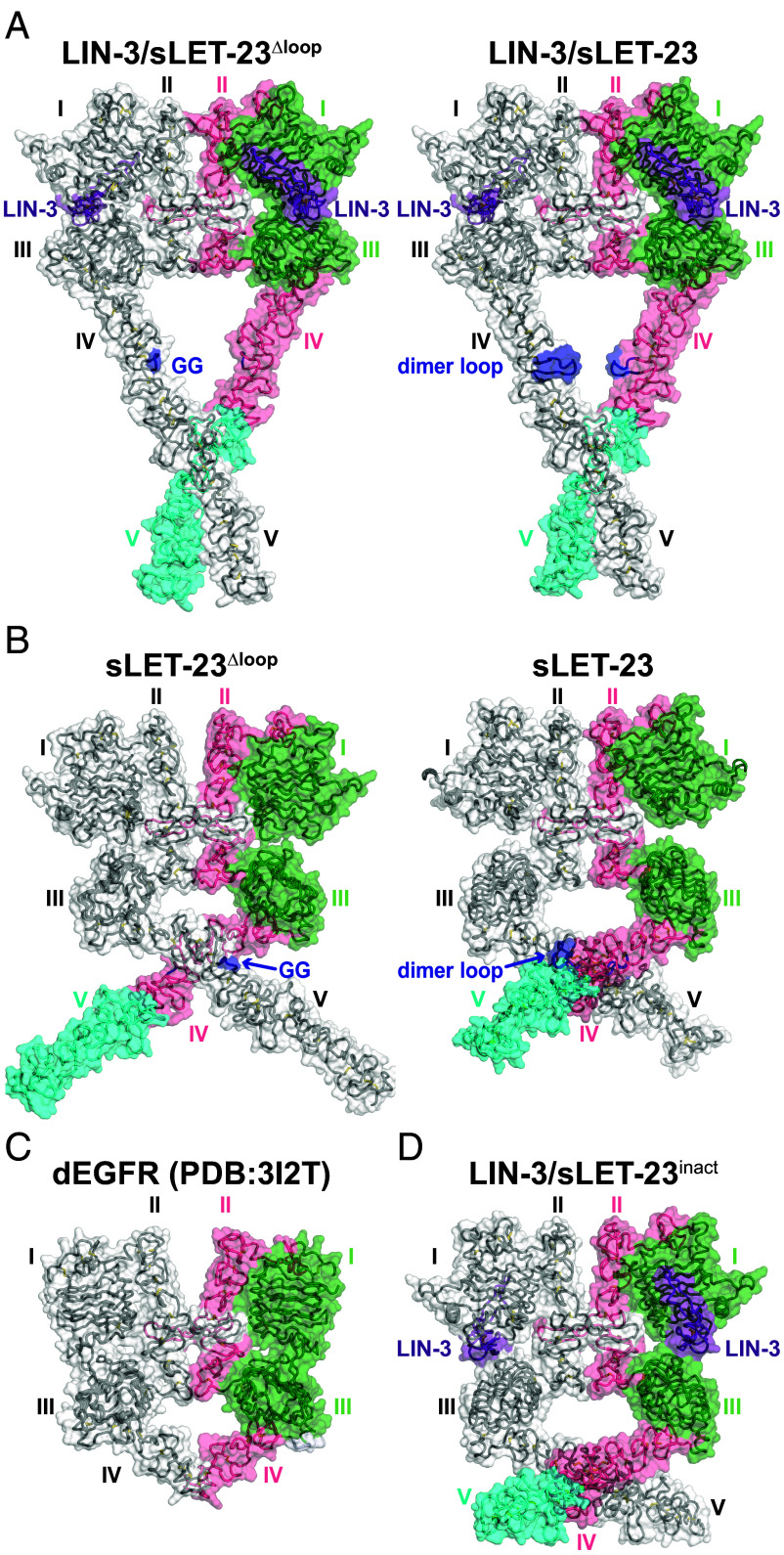
Alternate and competing states of sLET-23. Ribbon views with transparent molecular surfaces with domains colored as in Fig. 2*A*. (*A*) Deletion of the dIV dimer loop has no effect on the structure of the LIN-3/sLET-23 dimer. The LIN-3/sLET-23^Δloop^ complex is shown on the left along-side the structure of the WT protein (*Right*). The GG insert in sLET-23^Δloop^ (dark blue, *Left*) replaces the sLET-23 dIV dimer loop (dark blue, *Right*). (*B*) The weak unliganded dimer of sLET-23^Δloop^ (*Left*) is distinct from that of WT sLET-23 dimer (*Right*). The sLET-23^Δloop^ dimer is mediated largely by the dII dimer arm. The GG insert (dark blue) is on the outside dIV surface in the sLET-23^Δloop^ dimer whereas the dimer loop (dark blue) is buried in the dIV dimer interface sLET-23 dimer (*Right*). (*C*) The crystallographic dimer of the *D. melanogaster* EGFR (dEGFR, PDB:312 T) (51) resembles the unliganded sLET-23^Δloop^ dimer. In both complexes the two dIIs are straight and splayed apart. Domain V was not present in the crystalized dEGFR, and only part of dIV could be modeled (51). AlphaFold3 of dEGFR predicts relatively straight domains IV and V, similar to our sLET-23 structures (*SI Appendix*, Fig. S7*C*). (*D*) An inactive ligand-bound sLET-23 dimer where the dIV interface is retained and ligand-binding is compromised. Domain V is less well ordered in this structure and only the first four disulfide-bonded modules are modeled (to aa 731).

To exclude the possibility of major structural alterations in LET-23 with the dIV loop deletion, we determined a cryo-EM structure of sLET-23^Δloop^ with and without bound LIN-3. When bound to LIN-3, LET-23^Δloop^ forms very similar dimers to those seen with WT sLET-23 (Fig. 5*A* and *SI Appendix*, Figs. S5 *A*–*F* and S6*H*), consistent with the lack of dIV contributions to the ligand-bound dimer.

In the absence of ligand, analytical ultracentrifugation studies detected only monomers of sLET-23^Δloop^ (Fig. 4*B*). Since the protein concentrations used in that experiment allow us only to place a lower bound on dissociation constant (*K*_D_) of approximately 20 to 30 μM, we also performed cryo-EM studies at higher concentrations. These studies revealed unliganded sLET-23^Δloop^ dimers with multiple conformations (*SI Appendix*, Fig. S5 *G*–*M*). Dimerization is mediated primarily by the dII dimer arm, with very limited additional dII dimer contacts. Interestingly, the best-resolved reconstruction of the unliganded sLET-23^Δloop^ dimer (overall resolution ~ 4 Å; *SI Appendix*, Figs. S5 *M* and S6 *I*) closely resembles the crystallographic dimer of the unliganded ECR of *D. melanogaster* EGFR (51), with the two dII copies splayed apart compared with the arrangement seen for WT sLET-23 (Fig. 5 *B* and *C*). This presumably reflects a loss of dimerization strength resulting from removal of the dIV dimer loop (and its absence in the fly receptor). Moreover, the well-defined right-handed crossing of the rigid domain IV–V legs seen in WT sLET-23 is lost in sLET-23^Δloop^ (Fig. 5*B*). With the dIV dimer loop deleted, these legs no longer make intimate contact with one another. Although their trajectories are similar to those in WT sLET-23, they do not interact strongly, and cross in a left-handed rather than right-handed sense in most conformations. We suggest that the sLET-23^Δloop^ structure shown in Fig. 5*B* represents a plausible model for a preformed *D. melanogaster* EGFR dimer. Indeed, AlphaFold3 (52) models for the *Drosophila* receptor dimer very closely resemble the sLET-23^Δloop^ structure (*SI Appendix*, Fig. S7*C*). It also seems reasonable to argue that this dimer conformation might represent some preformed dimers of human EGFR and its relatives detected at the cell surface. For human EGFR family members, which lack dV, the C-terminal parts of the ECR adjacent to the membrane would be ~60 to 80 Å apart in such a hypothetical model, which should be sufficient to limit intracellular kinase domain dimerization and activation. Destabilization of this hypothetical preformed EGFR dimer could sensitize EGFR to ligand-induced activation, as we see for LET-23^Δloop^.

### Competing Sets of Interactions.

In analyzing our cryo-EM data for LIN-3/sLET-23 complexes (*SI Appendix*, Fig. S3), we were able to discern two mutually exclusive conformations, which have important implications for how different sets of interactions must compete and cooperate for LET-23 to be activated. The highest-resolution structure shown in Fig. 2*A* predominates when LIN-3/sLET-23 complexes were applied to grids after incubation at room temperature. In this conformation, interactions of LIN-3 with both domains I and III are optimized by domain reorientation (Fig. 3 *A* and *B*), increasing intimacy of the dII-mediated dimer interface but breaking the dIV dimer loop interactions seen in the absence of ligand. However, ~20% of particles in this dataset (*SI Appendix*, Fig. S3*F*) adopt an alternate conformation that has ligand bound (loosely), but more closely resembles the unliganded sLET-23 dimer. They retain the dIV dimer loop interactions seen without ligand (with associated separation of C-termini), have a less intimate dII dimer interface (with little dIII reorientation), and show LIN-3 bound primarily to dI (Fig. 5*D* and *SI Appendix*, Fig. S6*J*). Intriguingly, this alternative conformation was found to dominate in another LIN-3/sLET-23 complex dataset for which samples had not been exposed to higher temperatures before being placed on EM grids (*SI Appendix*, Fig. S6 *A*–*G*)—representing over 90% of particles.

These observations suggest that ligand-bound LET-23 explores alternative conformations with different sets of interactions dominate at the dimer interface. When dIV contacts dominate, the dII interface is constrained, and so is ligand binding (Fig. 5*D*); dIV loop interactions are autoinhibitory. When the dII interface dominates (which requires ligand binding), dIV interactions are broken, and the receptor’s intracellular kinase domains can come into close proximity (Fig. 5*A*).

Our sLET-23 structures reveal the conformational requirements for i) A ligand-bound active dimer (Fig. 5*A*); ii) A ligand-bound inactive dimer (Fig. 5*D*); and iii) An unliganded inactive dimer (Fig. 5*B*). Importantly, transitions between these conformations can all be described within the framework of ligand-induced changes in the human EGFR, for which only ligand-bound active dimers and inactive monomers have been described structurally.

### Unifying the EGFR and IR Activation Mechanisms.

Recent work with the structurally related IR family has focused on the same three classes of dimer described here for an EGF receptor (Fig. 2 *B* and *C*) and listed above. We previously highlighted the conceptual similarities between LET-23 and IR (5). Based on the structures described here, we would liken the dIV dimer loop-mediated interactions described here to the covalent association of IR α chains (plus the L2–FNIII-1 intermolecular interactions) as signaling restraints rather than a means to form preformed dimers required for signaling. Indeed, a monomeric insulin receptor, generated by disruption of the disulfide bonds linking the two α-chains, can still be activated by ligand (53)—indicating that the preformed dimer of IR is not required for receptor activation. Moreover, the disulfide-bonds that link the C-terminal regions of the insulin family receptor α-chains (α-CT) are important for modulating ligand responsiveness (54), but are not required for signaling per se—as we show for the dIV dimer loop in LET-23.

Our proposed mechanism for LET-23 regulation bears striking similarity to the mechanism proposed for the IR family (Fig. 2*C*) (26, 30, 31, 55–57). In both cases, preformed dimers hold the two intracellular kinase domains of a dimer apart so that they cannot trans-phosphorylate one another and the receptor remains inactive. Ligand binding induces conformational changes in the receptor dimers to break this constraint, allowing the kinase domains to come into close proximity and the receptor to become activated. Our structure of the unliganded sLET-23 dimer provides a structural view of a preformed EGFR dimer, a state that has long been proposed to be relevant in the human EGFR family (10–17). It also underlines long-appreciated structural and mechanistic parallels between the EGFR and IR families of RTKs. Indeed, early studies of these RTKs showed that chimeric receptors with swapped ECRs retained the functional characteristics of the intracellular region—EGF activated IR-like responses in a chimera with the EGFR ECR and IR intracellular region and vice versa (58, 59). Thus, understanding LET-23 provides a structural framework for understanding a unified activation mechanism for these two important RTK families and may represent an evolutionary missing link.

## Materials and Methods

### Cryo-EM Structure Determination.

The sLET-23, sLET-23^Δloop^ and LIN-3 were expressed and purified essentially as previously described (19) with modifications detailed in *SI Appendix*. For cryo-EM of WT LIN-3/sLET-23, samples with ~1.6-fold molar excess of ligand were frozen using samples stored on ice (Dataset S2) or with an additional incubation for ~1 h at room temperature (Dataset S1). Additional details of cryo-EM sample preparation, data collection, and structure determination for i) sLET-23 (*SI Appendix* Fig. S2), ii) LIN-3/sLET-23 Dataset S1 (*SI Appendix* Fig. S3), iii) LIN-3/sLET-23 Dataset S2 (*SI Appendix* Fig. S6), iv) sLET-23^Δloop^ (*SI Appendix F*ig. S5), and v) LIN-3/sLET-23^Δloop^ (*SI Appendix* Fig. S5), are described in *SI Appendix*. AlphaFold2 (60) prediction models of the LET-23 ECR and LIN-3 EGF domain were used as the initial models, and the best resolution model (2.4 Å LIN-3/sLET-23 complex) was built and refined first to facilitate model fitting and building for other datasets, as described in *SI Appendix*. Detailed parameters and statistics for the model building and refinement process are provided in *SI Appendix*, Table S1.

### Sedimentation Equilibrium Analytical Ultracentrifugation (SE-AUC).

Effects of domain IV alterations on sLET-23 dimerization with and without ligand were analyzed by SE-AUC experiments using an XL-I analytical ultracentrifuge (Beckman) exactly as described (19).

### Generation of *C. Elegans let-23* Mutants.

CRISPR-Cas9 and homology-directed repair were used to engineer alterations to the endogenous *let-23* locus to produce the following mutant genotypes: i) repair were used to engineer alterations to the endogenous *let-23* (*3A*) directing L559A + Q562A + M565A substitutions, ii) *let-23* (*4A*) directing L559A + N561A + Q562A + M565A substitutions, and iii) *let-23* (*Δloop*) directing the deletion of amino acids 594–602 plus insertion of two glycines. For each change, two independent mutants resulting from independent F_1_ progeny of animals injected with CRISPR reagents were preserved. See additional details in *SI Appendix*.

### Phenotypic Analysis.

Animals were grown at constant temperature from eggs or from developmentally arrested food-deprived first stage (L1) larvae. Animals (>50 for each group) were examined to assess their phenotypes using a dissecting microscope (Wild M5A; Leica Microsystems, Wetzlar, Germany). See additional details in *SI Appendix*.

The vulvaless phenotype caused by mutations in *lin-2* and *lin-3* was scored by assessing animals for the inability to lay eggs as detailed in *SI Appendix*. Time to adulthood was scored by placing developmentally arrested L1 larvae on plates with food and removing a random sample of fifty animals every 2 h to determine the proportion that were adults, as determined by the completion of vulval morphogenesis. Multivulval (Muv) phenotype was scored by growing animals either from eggs or from developmentally arrested L1 larvae at fixed temperatures and then scoring them roughly 1 d after they became adults for the presence of visible ectopic vulval tissue (“pseudovulvae” or “blips”), 50 animals per genotype per experiment. Animals were categorized as having no obvious ectopic pseudovulvae, as having one ectopic pseudovulva, or as having two or more ectopic pseudovulvae. Additional details in *SI Appendix*.

## Supplementary Material

Appendix 01 (PDF)

Dataset S01 (XLSX)

Dataset S02 (XLSX)

Dataset S03 (XLSX)

Dataset S04 (XLSX)

Dataset S05 (XLSX)

## Data Availability

The raw electron micrographs have been deposited in EMPIAR as EMPIAR-13588 (sLET-23) (61), EMPIAR-13583 (LIN-3/sLET-23, Dataset S1) (62), EMPIAR-13584 (LIN-3/LET-23, Dataset S2) (63), EMPIAR-13598 (sLET-23^Δloop^) (64), and EMPIAR-13597 (LIN-3/sLET-23^Δloop^) (65). The final cryo-EM maps have been deposited in the Electron Microscopy Data Bank under the accession numbers EMD-73275 (sLET-23) (66), EMD-73276 (LIN-3/sLET-23, from Dataset S1) (67), EMD-73277 (LIN-3/LET-23^inact^, from Dataset S2) (68), EMD-73278 (sLET-23^Δloop^) (69), and EMD-73279 (LIN-3/sLET-23^Δloop^) (70). The final models have been deposited in the Protein Data Bank (PDB) under accession codes 9YOR (sLET-23) (71), 9YOS (LIN-3/sLET-23, from Dataset S1) (72), 9YOT (LIN-3/sLET-23^inact^, from Dataset S2) (73), 9YOU (sLET-23^Δloop^) (74) and 9YOV (LIN-3/sLET-23^Δloop^) (75). Mutant strains have been cryopreserved and are available upon request from P.W.S. All other data are included in the manuscript and/or supporting information.
