# Supplementary material for: Ligand regulation and function of preformed EGFR dimers
Source: Proc Natl Acad Sci U S A. 2026 Jul 20;123(30):e2602436123. doi: 10.1073/pnas.2602436123 (PMC13416820; doi:10.1073/pnas.2602436123)
Supplement: Supplementary file 1 — Appendix 01 (PDF) [file pnas.2602436123.sapp.pdf]

PNAS

Supporting Information for
Ligand regulation and function of preformed EGFR dimers

Yuhong Zuo, Hillel T. Schwartz, Kahlil Walker, Long Han, Paul W. Sternberg & Kathryn M. Ferguson*

***Corresponding author**: Kathryn M. Ferguson
Email: kathryn.ferguson@yale.edu

This PDF file includes:

Extended Methods
Figures S1 to S7
Tables S1 to S3
Legends for Datasets S1 to S5
SI References

Other supporting materials for this manuscript include the following:

Datasets S1 to S5

Supporting Information Text

Extended Methods

Insect cells

Spodoptera frugiperda Sf9 cells were propagated at 27º C with constant shaking at 120 rpm in ESF 921 serum-free insect cell culture medium (Expression Systems), supplemented with 50 U/mL penicillin/streptomycin. *D. melanogaster* Schneider 2 (S2) cells were maintained at 27º C in Schneider's Insect Medium (Sigma-Aldrich) supplemented with 10% fetal bovine serum. For protein expression, stably transfected S2 cell pools were grown at 27º C in ESF-921 Insect Cell Culture Medium (Expression Systems) or EX-CELL 420 Serum Free Medium (Sigma-Aldrich) in the presence of 200 mg/mL hygromycin-B.

C. elegans strain maintenance and genetics

C. elegans strains were derived from the wild-type strain N2 (Bristol) and cultured according to standard conditions (1). Mutations used that were not generated in the course of this study include: *lin-53(n2978), let-23(sa62), lin-36(n766), lin-3(n378), lin-2(n105), lin-15A(n433), lin-15A(n2375), lin-15AB(n765),* and *lin-15AB(n2993 n433)* (2-5). Balancer chromosomes used include *mIn1 [dpy-10(e128) mIs14], mIn1 [dpy-10(e128) umnIs43],* and *tmC9 [F36H1.2(tmIs1221)]* (6, 7). Strains were constructed by using passage through males to homozygous the X chromosome, on the basis of their mutant phenotype, or by following mutations of interest in *trans* using balancer chromosomes. All animals examined were hermaphrodites.

Protein expression and purification

The full-length extracellular region of the *C. elegans* EGFR LET-23 (sLET-23, amino acids [aa] 1–819 including the 26 aa native signal peptide) was described previously (8). To generate sLET-23$^{\Delta loop}$, the pFastBac-1 plasmid encoding sLET-23 was modified so that the expressed protein has a GG dipeptide in place of 9 amino acids in domain IV ($_{594}$YNSDFGNRM^{602}). The alteration was confirmed by DNA sequencing.

The sLET-23 and sLET-23$^{\Delta loop}$ proteins were expressed in Sf9 cells using recombinant baculoviruses essentially as described (8) with the exception that transfection of bacmid DNA into Sf9 cells used linear polyethyleneimine (PEI) (9). The LIN-3 EGF domain (residues K148–N206, with numbering from isoform F36H1.4c on WormBase) was expressed as a secreted fusion protein with residues 44–76 of *D. melanogaster* SPITZ (LIN-3^{xEGF}) (10) in stable S2 cells as described previously (8). Clarified conditioned media was passed over a Ni-Penta (Marvelgent Biosciences), washed with 1x Phosphate-Buffered Saline, pH 7.4 (PBS buffer) and the bound protein eluted stepwise with increasing concentrations of imidazole in PBS buffer. For cryo-EM with WT sLET-23, protein-containing fractions were pooled, concentrated and used without further purification. Alternatively, conditioned media was first buffer exchanged into 25 mM Tris, 150 mM NaCl, pH 8.0 (Buffer A) using a 30 kDa molecular weight cutoff Sartocon Slice ECO diafiltration cassette and then applied to a Ni-NTA (Thermo Scientific) affinity column. The bound protein was eluted stepwise with increasing concentrations of imidazole in Buffer A. For sLET-23, protein-containing fractions were concentrated and further purified on a Superose 6 Increase 10/300 GL column (GE Healthcare) equilibrated with 25 mM HEPES, 150 mM NaCl, pH 8.0 (Buffer B) or PBS buffer. For biophysical analysis, LIN-3^{xEGF} was treated with Factor Xa and the EGF domain (LIN-3^{EGF}) purified exactly as described (8).

Cryo-EM sample preparation and data collection

Unliganded sLET-23 (~10 mg/mL) in PBS buffer was used directly for grid preparation. The LIN-3/sLET-23 complex was prepared by mixing equal volumes of purified sLET-23 (~10 mg/mL) and LIN-3^{xEGF} proteins (~2 mg/mL) (molar ratio ~1:1.6) on ice with or without additional incubation for ~1 hour at room temperature. For sLET-23$^{\Delta loop}$ protein in buffer B at 6 mg/mL was used directly for grids or mixed with a 0.5 molar ratio of LIN-3^{EGF}. All cryo-EM grids were prepared using a Vitrobot Mark IV (Thermo Fisher Scientific) as follows: 3 μL of protein was applied to glow-discharged Quantifoil holey carbon grids (R1.2/1.3 or R2/1 300 mesh, gold or copper), blotted with filer paper for 5–10 s in > 90 % humidity at 16º C, and plunge frozen in liquid ethane cooled by

liquid nitrogen. Grids were screened for ice thickness and particle distribution using a Glacios (200 kV; Thermo Scientific) in the Yale CryoEM Resource. For WT sLET-23 and unliganded sLET-23$^{\Delta loop}$, images were collected on a Titan Krios microscope equipped with a K3 summit direct electron detector (Gatan) and a GIF quantum energy filter (15 eV) at the Laboratory for Biomolecular Structure, Brookhaven National Laboratory (LBMS/BNL). Images were recorded using EPU (Thermo Fisher Scientific) in super-resolution mode at a nominal magnification of 105,000×, corresponding to a calibrated pixel size of 0.825 Å on the specimen level and 0.413 Å for super-resolution images, with a preset defocus range from 0.8 to 2.0 µm. The dose rate was set to 15 electrons per physical pixel per second. The exposure time of each movie was 2.3 s, leading to a total dose of 50 electrons per Å^2, fractionated into 40 frames. Data for the LIN-3/sLET-23$^{\Delta loop}$ complex were collected at the Yale CryoEM Resource on a Glacios microscope equipped with a summit direct detection camera (Gatan) K3 camera. Automated data acquisition was performed using SerialEM 4.1 or 4.2 (11). Micrographs were collected at a 45000× magnification with a calibrated pixel size of 0.868 Å (super-resolution pixel size 0.434 Å) and a defocus range from approximately 0.8 to 2.0 µm. The electron dose was 40 electrons per Å^2, fractionated across 40 frames.

Cryo-EM image processing and 3D reconstruction

Data were processed with cryoSPARC (12). Motion-correction and CTF estimation were first carried out for all images. Micrographs with a CTF estimation resolution worse than 4.5 Å were excluded. After blob picking and several rounds of 2D classification, 2D classes with clear structural features were selected and used for template picking. Several rounds of 2D classification were performed, and the well-aligned 2D classes were selected to train a Topaz model (13), which was then used to pick particles from all images. After 2–3 rounds of 2D classification of the Topaz picked particles to discard junk and other poorly aligned particles, remaining particles were used for 3D reconstruction analysis. *Ab initio* model reconstruction was used to generate initial 3D models. Particles corresponding to different classes were selected and optimized through iterative rounds of heterogeneous refinement in cryoSPARC, without applying any symmetry restraint (C1 symmetry). The best models were refined using non-uniform refinement and local refinement. In all cases, the class maps display highly symmetrical features in early analyses, and C2 symmetry was applied in later rounds of heterogenous refinements and non-uniform refinements. The overall resolutions were estimated based on the gold-standard Fourier shell correlation (FSC) = 0.143 criteria. Local resolution was estimated from two half data maps under cryoSPARC. Specific details for each dataset are as follows:

i). sLET-23 (Fig. S2): 5,200,000 particles were extracted from 10,000 micrographs, following the Topaz training, and 2,900,000 selected after two rounds of 2D classification. *Ab initio* reconstruction of ~600,000 particles was performed with six classes specified, resulting in six distinct density maps, each representing a unique structural conformation. All six maps were subjected to heterogeneous refinement, and four high-quality class maps were subsequently selected for further analysis by non-uniform refinement.

ii). LIN-3/sLET-23 dataset 1 (Fig. S3): Processing followed as for sLET-23 dataset with 4,300,000 Topaz-picked particles from 8,777 micrographs reduced to 1,400,000 particles after 2D classification. Local refinement using a mask covering domain V and part of domain IV (amino acids 606–810) was also performed to generate an improved map of the dV/dV interactions in the active complex.

iii). LIN-3/sLET-23 dataset 2 (Fig. S6): Processing followed as for sLET-23 with 3,300,000 Topaz-picked particles from 5,497 micrographs reduced to 1,600,000 after 2D classification.

iv). sLET-23$^{\Delta loop}$ (Fig. S5): Processing followed as for sLET-23 dataset with 7,100,000 Topaz-picked particles from 30,967 micrographs reduced to 3,900,000 particles after 2D classification. Five high-quality class maps were selected for further analyses.

v). LIN-3/sLET-23$^{\Delta loop}$ (Fig. S5): Processing followed as for sLET-23 dataset with 1,600,000 Topaz-picked particles from 8,362 micrographs reduced to 709,000 particles after 2D classification. A single interpretable class arose from *ab initio* reconstruction that closely resembled the LIN-3/sLET-23 dimer shown in Fig. 2*A*. After several rounds of heterogeneous refinements and additional particle curations through 2D-classification, 148,822 particles are subject to non-uniform and local refinement to generate a final model at 3.36 Å.

Model building, refinement and structural analysis

For model building, DeepEMhancer (14) was employed to modify and sharpen the density maps. AlphaFold2 (15) prediction models of the LET-23 ECR and LIN-3 EGF domain were used as the initial models, and the best resolution model (2.4 Å LIN-3/sLET-23 complex) was built and refined first to facilitate model fitting and building for other datasets. The corresponding initial models were fit into the cryo-EM density maps using UCSF ChimeraX,(16) and the models were manually adjusted in Coot (17, 18). Real space refinements were performed in PHENIX (19). Detailed parameters and statistics for the model building and refinement process are provided in Table S1. Structural figures were generated in PyMOL (20) and UCSF ChimeraX (16). Analysis of molecular contacts and buried surface areas were done using UCSF ChimeraX (16) or the PDBePISA server (21). DynDom (22) was used to define conformational transitions between LET-23 states. AlphaFold models were generated with ColabFold (23) or the AlphaFold Server (24).

Sedimentation equilibrium analytical ultracentrifugation (SE-AUC)

Effects of domain IV alterations on sLET-23 dimerization with and without ligand were analyzed by SE-AUC experiments using an XL-I analytical ultracentrifuge (Beckman) exactly as described (8). Samples of wild-type and mutated sLET-23 ranging from 2 – 10 μM in Buffer B, with and without addition of 1.2-fold molar excess of LIN-3 were spun at 20º C at three speeds (between 5,000 and 10,000 rpm) in a An Ti 60 rotor. Radial absorption data were collected at 280, 285 and 290 nm. The resulting nine data sets (three speeds and three concentrations) were fit globally to determine the K_D^{dim} and / or ideal molecular mass using the program HeteroAnalysis (v1.1.60, written by J. Cole and J. Lary, University of Connecticut). The monomer molecular mass was assumed to be ~100 kDa based on the observation of seven N-glycosylation sites in the cryo-EM maps compared to the six that are predicted (8). The partial specific volume of sLET-23 was estimated as 0.71 mLg^{-1}. Wild-type sLET-23 and all samples with LIN-3 were essentially fully dimeric under these assay conditions (K_D^{dim} values in the sub-micromolar range). For sLET-23$^{\Delta loop}$, a lower limit for the K_D^{dim} was estimated to be 20–30 μM. Data at each radial positions are plotted in Fig. 4*B* as $(r^2-r_0^2)/2$, where r_0 is the meniscus. Data are representative of at least three independent experiments using different protein preparations (*Dataset S1*).

Generation of *C. elegans let-23* mutants

CRISPR-Cas9 and homology-directed repair were used to introduce to the endogenous *let-23* locus selected changes in extracellular domain IV, intended to reduce dimerization: L559A + Q562A + M565A (for which the mutant genotype will be labeled as "*let-23(3A)*"), L559A + N561A + Q562A + M565A (for which the mutant genotype will be labeled as "*let-23(4A)*"), and the deletion of amino acids 594–602 together with the insertion at that site of two glycines (for which the mutant genotype will be labeled as "*let-23(Δloop)*"). Genome editing was made more efficiently detectable by co-conversion at *unc-58* as described (25). Single-stranded DNA oligonucleotides were used to direct the repair and had at least 40 nucleotides of homology on each side flanking the cleavage site and nucleotide changes. In addition to the intended coding change, additional changes disrupted homology to the guide RNA, disrupted the NGG PAM motif, and either generated or disrupted restriction enzyme cleavage sites; all additional changes were selected to make no coding change, and all changes were designed to avoid infrequently used codons (26). Changes were confirmed by PCR amplification using oligonucleotide primers with the sequences TGTCGAAAATTGATTGGTCATC and GACGGTAGCTAAAATCATTCGT followed by Sanger sequencing (Laragen, Culver City, CA). DNA oligonucleotides, tracrRNA, and sequence-specific crRNAs were synthesized by Integrated DNA Technologies (Coralville, IA). For each designed change, two independent mutants resulting from independent F_1 progeny of animals injected with CRISPR reagents were preserved. Sequences used in the generation of crRNAs and oligonucleotide repair template sequences and the identifying allele numbers of the resulting *let-23* mutations are listed in Tables S2 and S3.

Phenotypic analysis

Animals were grown at constant temperature from eggs or from developmentally arrested food-deprived first stage (L1) larvae. Eggs were picked from plates by hand or were recovered by

dissolving their gravid mothers in sodium hydroxide with sodium hypochlorite (0.8M NaOH, 0.25 % available chlorine) and rinsing them twice with water, before either placing the eggs directly on NGM plates with lawns of OP50 bacteria as a food source or allowing the eggs to hatch in S medium in the absence of food and enter developmental arrest, before placing the L1 larvae on plates with food to resume development. Animals (> 50 for each group) were examined to assess their phenotypes using a dissecting microscope (Wild M5A; Leica Microsystems, Wetzlar, Germany). Data resulting from phenotypic analysis are presented as the mean ± standard deviation (*Datasets S2 and S3*).

The vulvaless phenotype caused by mutations in *lin-2* and *lin-3* was scored by assessing animals for the inability to lay eggs: developmentally arrested L1 larvae were placed on NGM plates containing OP50 as food and allowed to grow at 20º C for 44–48 hours, until they reached the late L4 (fourth stage larval) stage of development. For each genotype, 40 L4 larvae were transferred to each of three new NGM Petri plates that had lawns of OP50 as a food source. These plates were checked after approximately 24 hours for the inability of the animals to lay eggs, demonstrated by whether they had retained an unusually large number of eggs. Time to adulthood was scored by placing developmentally arrested L1 larvae on plates with food at 20º C or at 18.5º C and allowing them to grow. Every two hours a random sample of fifty animals was examined to determine the proportion that were adults, as determined by the completion of vulval morphogenesis (*Dataset S4*).

Multivulval (Muv) phenotype was scored by growing animals either from eggs or from developmentally arrested L1 larvae at fixed temperatures and then scoring them roughly one day after they became adults for the presence of visible ectopic vulval tissue ("pseudovulvae" or "blips"), 50 animals per genotype per experiment. Animals were categorized as having no obvious ectopic pseudovulvae, as having one ectopic pseudovulva, or as having two or more ectopic pseudovulvae. In the case of *lin-15AB(n765)*, which showed a nearly fully penetrant multivulval phenotype when grown from arrested L1 larvae even at 15º C, fifty animals of each genotype were arbitrarily assessed for the size of their pseudovulval blips (small or large) (*Dataset S5*).

For all *C. elegans* experiments, sample sizes were set to at least 50 to enable efficient data collection and random, non-exhaustive sampling from larger populations while providing sufficient statistical power for the comparisons being made. Animals were randomly selected for analysis from a larger population. Errors are the standard deviation on the mean. P-values were determined by Fisher's Exact Test.

Figures

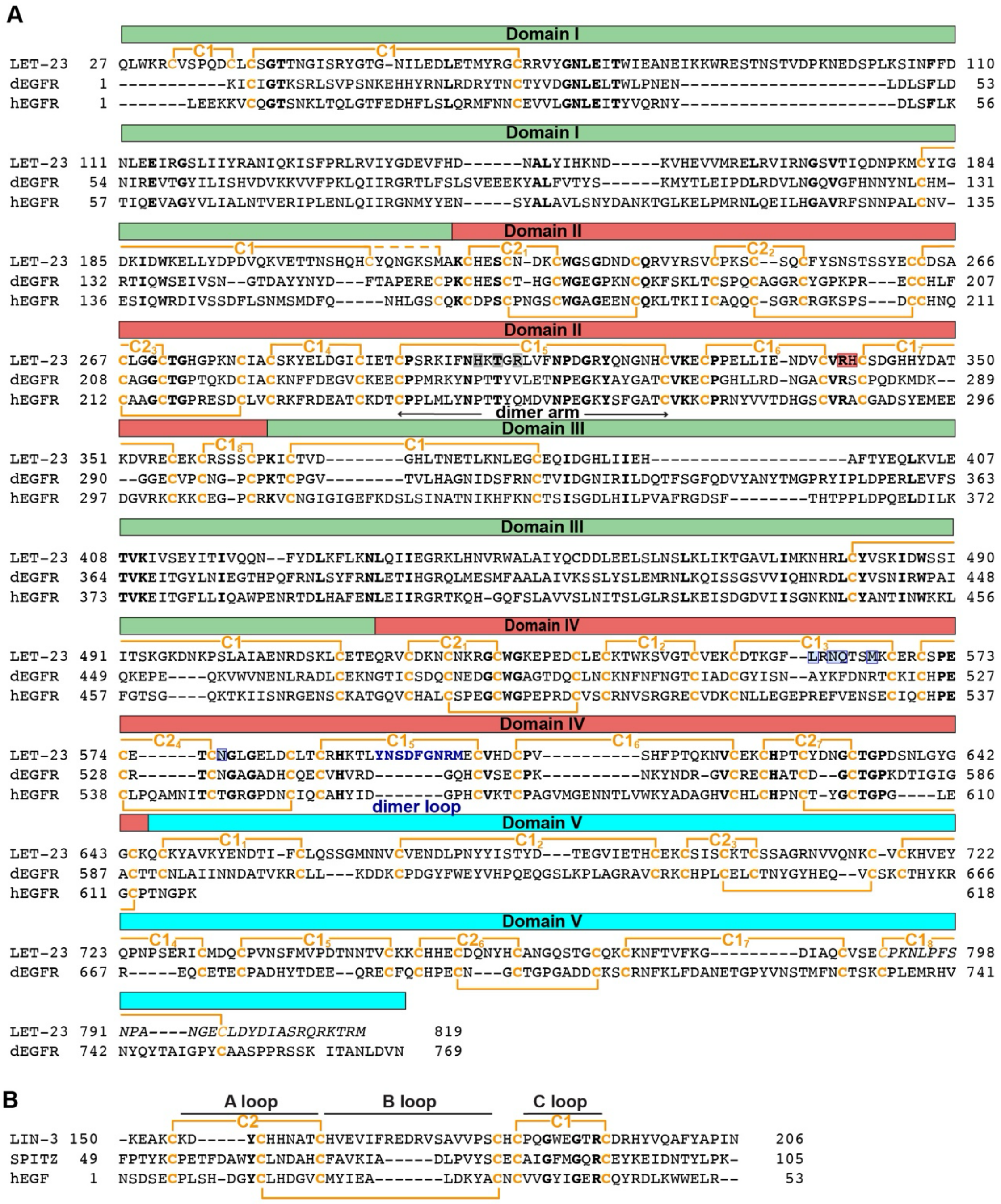

Fig. S1. Sequence comparison of EGFR ECRs from *C. elegans* (LET-23), *D. melanogaster* (dEGFR) and *H. sapiens* (hEGFR), and representative ligands.

(*A*) A structure-based sequence alignment of the ECRs of LET-23, dEGFR and hEGFR. Alignments were guided by available structures (sLET-23 this study, PDB:9YOR; dEGFR, PDB:3I2T; and hEGFR, PDB:3NJP), supplemented with AlphaFold2 (15) predictions for missing regions (including the dEGFR dV). Domains are highlighted with a colored bar over the sequence (I and III in green, II and IV in salmon and V in cyan). Where there is identity across all three proteins the amino acid letter is in bold. Cysteines are orange and disulfide bonding is indicated. The disulfide-bonded

modules are classified as C1 (single disulfide) or C2 (two disulfide knot), and numbered sequentially in each domain (II, IV and V). The dII dimer arm is indicated and amino acids involved in dII dimerization highlighted with colors as in Figs. 1*C* and 3*C* (grey in the dimer arm and salmon in the other protomer interacting with that dimer arm). The dIV dimer loop is colored dark blue and the amino acids that were altered to weaken the dIV dimer interactions are highlighted in blue. The structure of a dV has not previously been determined experimentally. The pattern of disulfide-bonded modules in dV comprises two C1–C1–C2 repeats, as seen in dIV (excluding the first dIV C2 module that is structurally integral to dIII), and ends with two C1 modules. In other invertebrate EGFR family receptors that have extended membrane proximal cysteine rich regions, the pattern of repeating C1–C1–C2 modules appears to be conserved. Amino acids are numbered from the start of the signal peptide for LET-23 and from the start of the mature protein for dEGFR and hEGFR, for consistency with published structures. Density after the dV disulfide-bonded module $C1_7$ is weak in all reconstructions and aa 792-819 (italic text) are not included in the structure figures. (*B*) Sequence alignments of the EGF domain of LIN-3 with representative ligands of dEGFR (SPITZ) and hEGFR (EGF). Disulfide bonding is show as in *A* and the structural loops (A, B and C) are indicated.

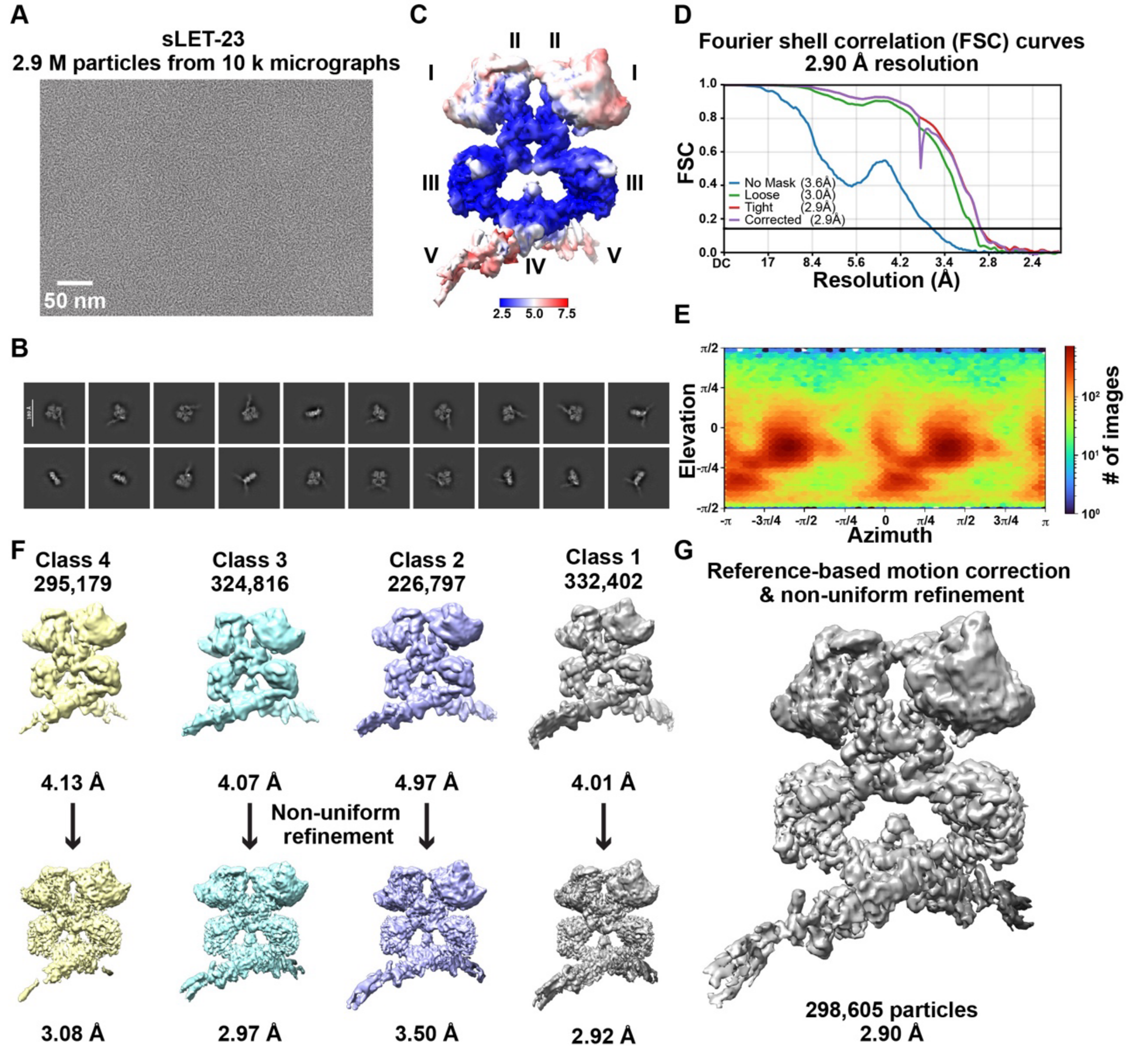

Fig. S2. Cryo-EM data processing of the unliganded sLET-23 dataset.
(*A*) A representative motion-corrected micrograph of the unliganded sLET-23. The number of micrographs and particles selected for three-dimensional reconstruction analysis are indicated. (*B*) Example two-dimensional class averages of particles selected for further processing. (*C*) Local resolution estimation shown on the sLET-23 density map. (*D*) Gold Standard Fourier Shell Correlation (GSFSC) for the final cryo-EM map used for model building with a reported resolution of 2.9 Å. (*E*) Angular distribution plot of particles used in final non-uniform refinement. (*F*) Details of the four 3D classes selected from heterogeneous refinements for further analysis. Number of particles are indicated along with the resolution before and after non-uniform refinement. (*G*) Final refined map for the most populated state after reference-based motion correction and non-uniform refinements. The total number of particles used to generate this map are indicated.

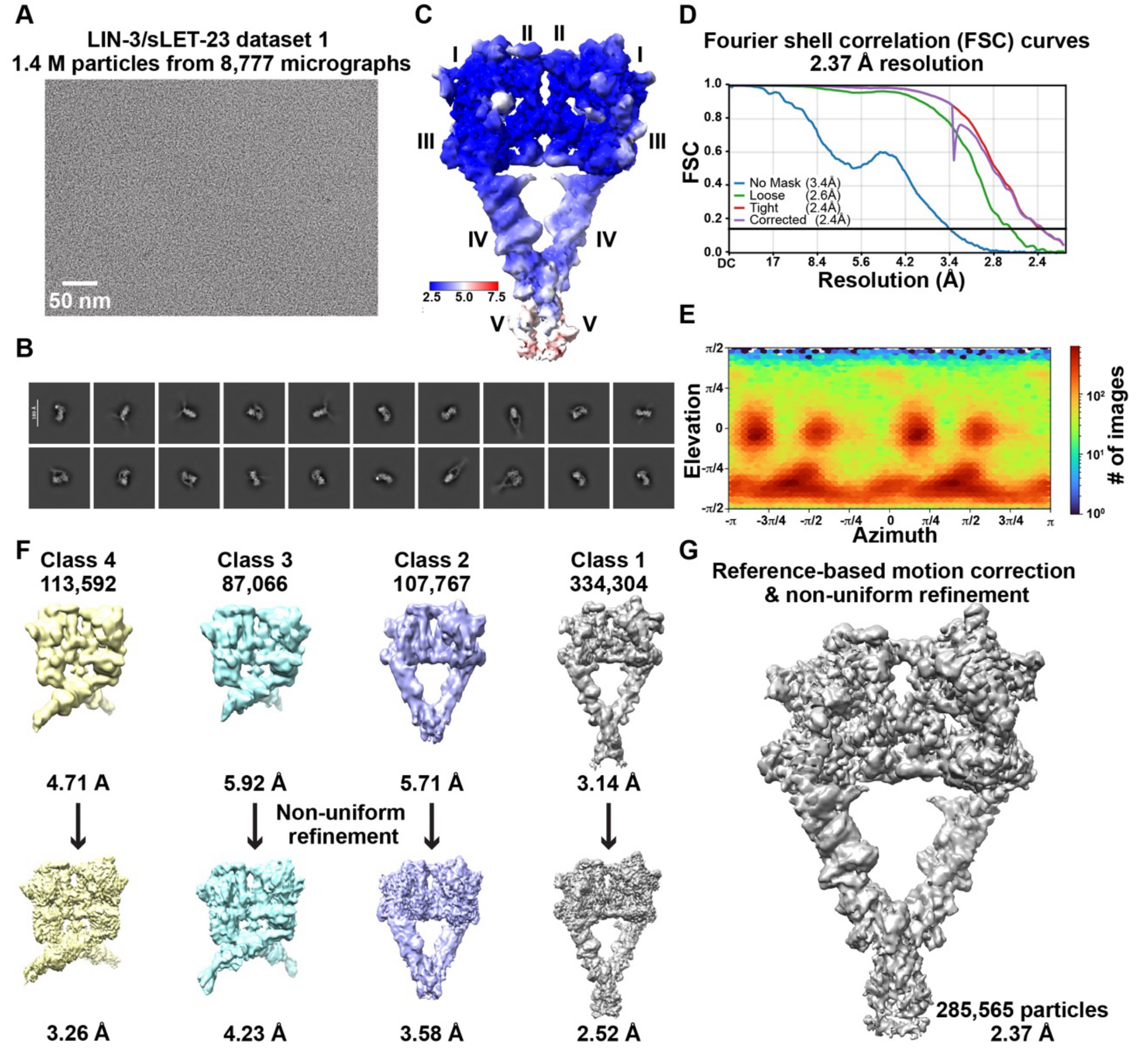

Fig. S3. Cryo-EM data processing of LIN-3/sLET-23 dataset 1.
(*A*) A representative motion-corrected micrograph of the LIN-3/sLET-23 dataset 1. The number of micrographs and particles selected for three-dimensional reconstruction analysis are indicated. (*B*) Example two-dimensional class averages of particles selected for further processing. (*C*) Local resolution estimation shown on the LIN-3/sLET-23 density map. (*D*) Gold Standard Fourier Shell Correlation (GSFSC) for the final cryo-EM map used for model building with a reported resolution of 2.37 Å. (*E*) Angular distribution plot of particles used in final non-uniform refinement. (*F*) Details of the four 3D classes selected from heterogeneous refinements for further analysis. Number of particles are indicated along with the resolution before and after non-uniform refinement. (*G*) Final refined map for most populated state after reference-based motion correction and non-uniform refinements. The total number of particles used to generate this map are indicated.

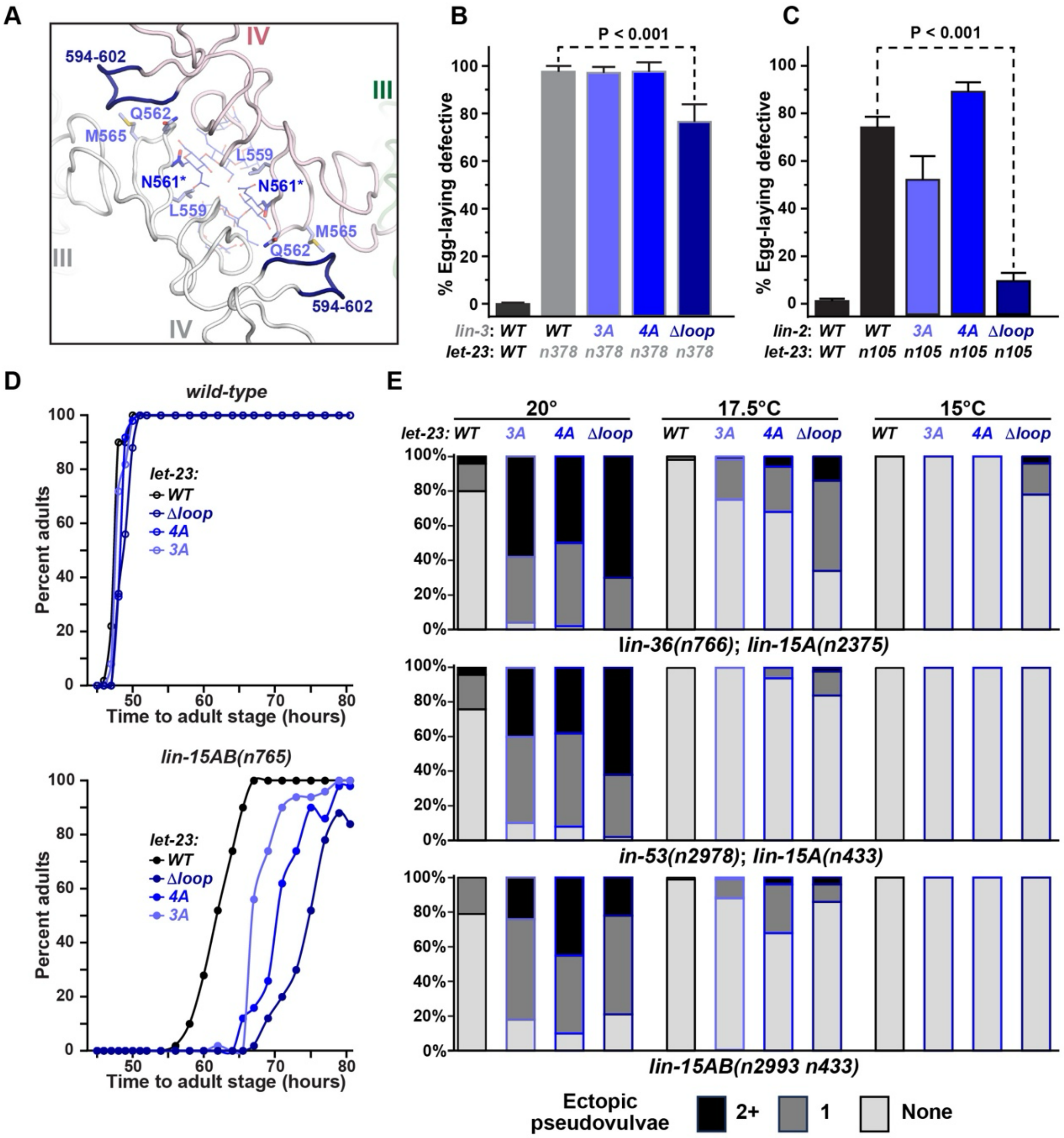

Fig. S4. Domain IV mutations increase LET-23 sensitivity to LIN-3.
(*A*) A view of the dIV dimerization interface similar to that shown in Fig. 4*A*, highlighting the alterations made to weaken the dimer interface. The loop in dark blue (aa 594–602) was replaced with two glycines (Δloop), L559, Q562 and M565 were mutated to alanine (3A) or L559, N561, Q562, and M565 mutated to alanine (4A). N561 (marked with an asterisk) is an N-linked glycosylation site. (B and C), Effect of dIV mutations on the Vul phenotype caused by loss-of-function alleles in the *lin-3-let-23* signaling axis, as determined by inability to lay eggs. Data shown in Fig. 4 *C* and *D* for the dIV loop deletion mutation (*Δloop*) are shown alongside data for the dIV alanine substitution mutations (*3A* and *4A*). Values are the mean from at least three experiments, each with n = 50. Error bars are standard deviation. (*B*) In the *lin-3(n378*) background, *let-23(3A)* and *let-23(4A)* have no effect on the Vul phenotype (*Dataset S2*). (*C*) In the *lin-2(n105)* background modest effect are seen, with *let-23(3A)* reducing the percentage unable to lay eggs to 52 ± 10% whereas *let-23(4A)* increases it to 89 ± 4% (*Dataset S3*). (*D*) Effects of the dIV mutations on the same developmental delay assay as presented in Fig. 4*E* (see legend to Fig. 4 for details). None of the mutations has effects in a wild-type background (upper panel, open circles). In *lin-15AB(n765)* mutant animals the delay in development is extended by addition of the dIV

mutations (lower panel, closed circles) by 25 % for the loop mutant (*Δloop*), and 5 and 12 % respectively for the three and four alanine mutations (*3A* and *4A*). *let-23* genotype is indicated as in *B* (*Dataset S4*). (*E*) Effects of weakening dIV interactions on the weakly penetrant and expressive multivulva (Muv) phenotypes in temperature sensitive synMuv mutants. Data are presented exactly as in Fig. 4*F*, here including the data for the alanine substitution mutants, *3A* and *4A*. Percentage of animals with no ectopic pseudovulvae (light gray), one ectopic pseudovulva (dark gray), or with two or more ectopic pseudovulvae (black) are shown; n = 50. Animals were grown at 20º C, 17.5º C, or 15º C. Three different synMuv backgrounds were tested: *lin-36(n766)*; *lin-15A(n2375)* (upper), *lin-53(n2978)*; *lin-15A(n433)* (middle), and *lin-15AB(n2994 n433*) (lower). *let-23* genotype is indicated as in (B) (*Dataset S5*).

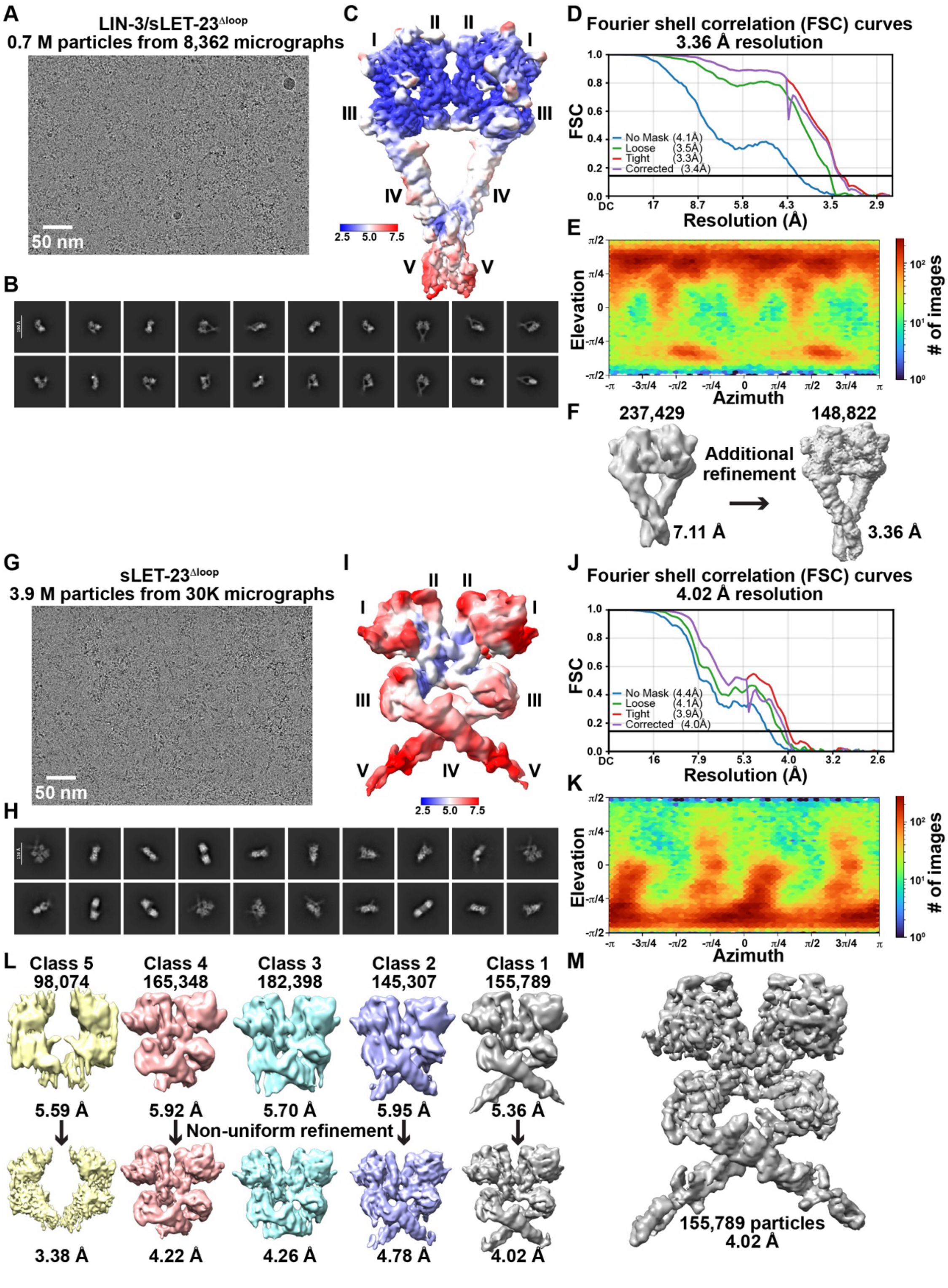

Fig. S5. Cryo-EM data processing of sLET-23$^{\Delta loop}$ with and without LIN-3.
(*A-F*) Cryo-EM data processing of LIN-3/sLET-23$^{\Delta loop}$ dataset. (*A*) A representative motion-corrected micrograph of the LIN-3/sLET-23$^{\Delta loop}$. The number of micrographs and particles selected for three-dimensional reconstruction analysis are indicated. (*B*) Example two-dimensional class averages of particles selected for further processing. (*C*) Local resolution estimation shown on the

LIN-3/sLET-23$^{\Delta loop}$ density map. (*D*) Gold Standard Fourier Shell Correlation (GSFSC) for the final cryo-EM map used for model building with a reported resolution of 3.36 Å. (*E*) Angular distribution plot of particles used non-uniform refinement. (*F*) The single 3D class used in heterogeneous refinements. Number of particles and resolution are indicated. Following additional refinement (see methods), 148,882 particles were used in the final refinement to 3.36 Å resolution.

(*G-M*) Cryo-EM data processing of the unliganded sLET-23$^{\Delta loop}$ dataset. (*G*) A representative motion-corrected micrograph of the sLET-23$^{\Delta loop}$. The number of micrographs and particles selected for three-dimensional reconstruction analysis are indicated. (*H*) Example two-dimensional class averages of particles selected for further processing. (*I*) Local resolution estimation shown on the sLET-23$^{\Delta loop}$ density map. (*J*) Gold Standard Fourier Shell Correlation (GSFSC) for the final cryo-EM map used for model building with a reported resolution of 4.02 Å. (*K*) Angular distribution plot of particles used final refinement. (*L*) Details of the five 3D class averages used in heterogeneous refinements. Number of particles are indicated along with the resolution before and after non-uniform refinement. (*M*) Final sharpened map (DeepEMhancer[65]) for Class 1. Resolution and number of particles are indicated.

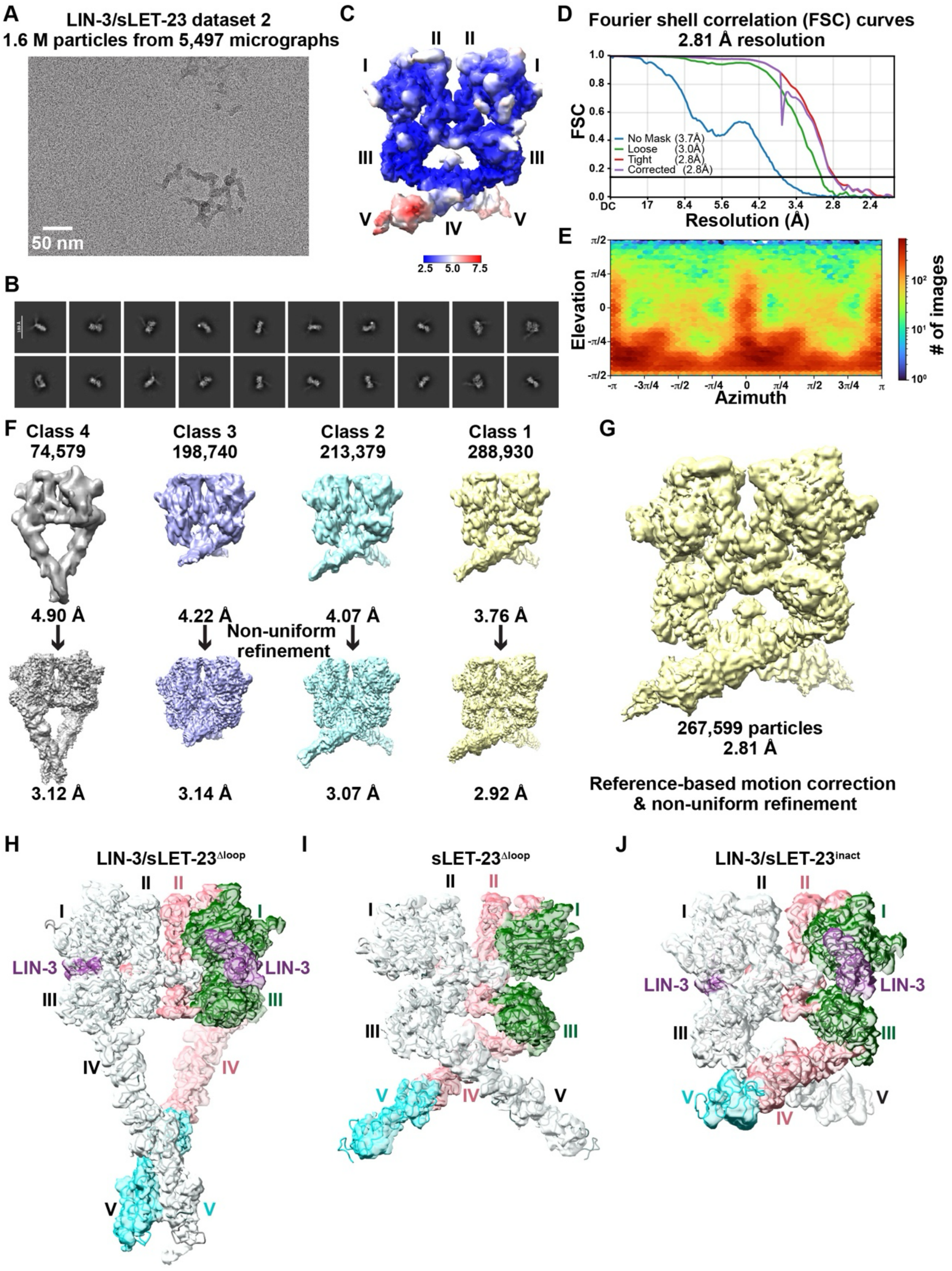

Fig. S6. Additional Cryo-EM structures

(*A*-*G*) Cryo-EM data processing and structure of LIN-3/sLET-23^{inact} from dataset 2 (*A*) A representative motion-corrected micrograph of the LIN-3/sLET-23 dataset 2. The number of micrographs and particles selected for three-dimensional reconstruction analysis are indicated. (*B*) Example two-dimensional class averages of particles selected for further processing. (*C*) Local resolution estimation shown on the LIN-3/sLET-23 dataset 2 density map. (*D*) Gold Standard

Fourier Shell Correlation (GSFSC) for the final cryo-EM map used for model building with a reported resolution of 2.81 Å. (*E*) Angular distribution plot of particles used final non-uniform refinement. (*F*) Details of the four 3D classes selected from heterogeneous refinements for further analysis. Number of particles for each class are indicated, along with the resolutions before and after non-uniform refinement. (*G*) Final refined map for most populated state in dataset 2 after reference-based motion correction and non-uniform refinements. 267,599 particles were used to generate this map. LIN-3/sLET-23^{inact} was structure was built and refined based on this map from dataset 2. (*H*-*J*) Cryo-EM maps of additional LET-23 structures. (*H*) Cryo-EM map and the resulting structural model for LIN-3 complex with sLET-23$^{\Delta loop}$, colored as in Fig. 2*A*. (*I*) Cryo-EM map and the resulting structural model for the weak unliganded dimer of sLET-23$^{\Delta loop}$, colored as in Fig. 2*A.* (*J*) Cryo-EM map and the resulting structural model for presumed inactive LIN-3/sLET-23^{inact} complex, colored as in Fig. 2*A*. Domain V is less well ordered in this structure compared to the unliganded sLET-23 dimer. Only the first four disulfide-bonded modules are modelled (ending at aa 731).

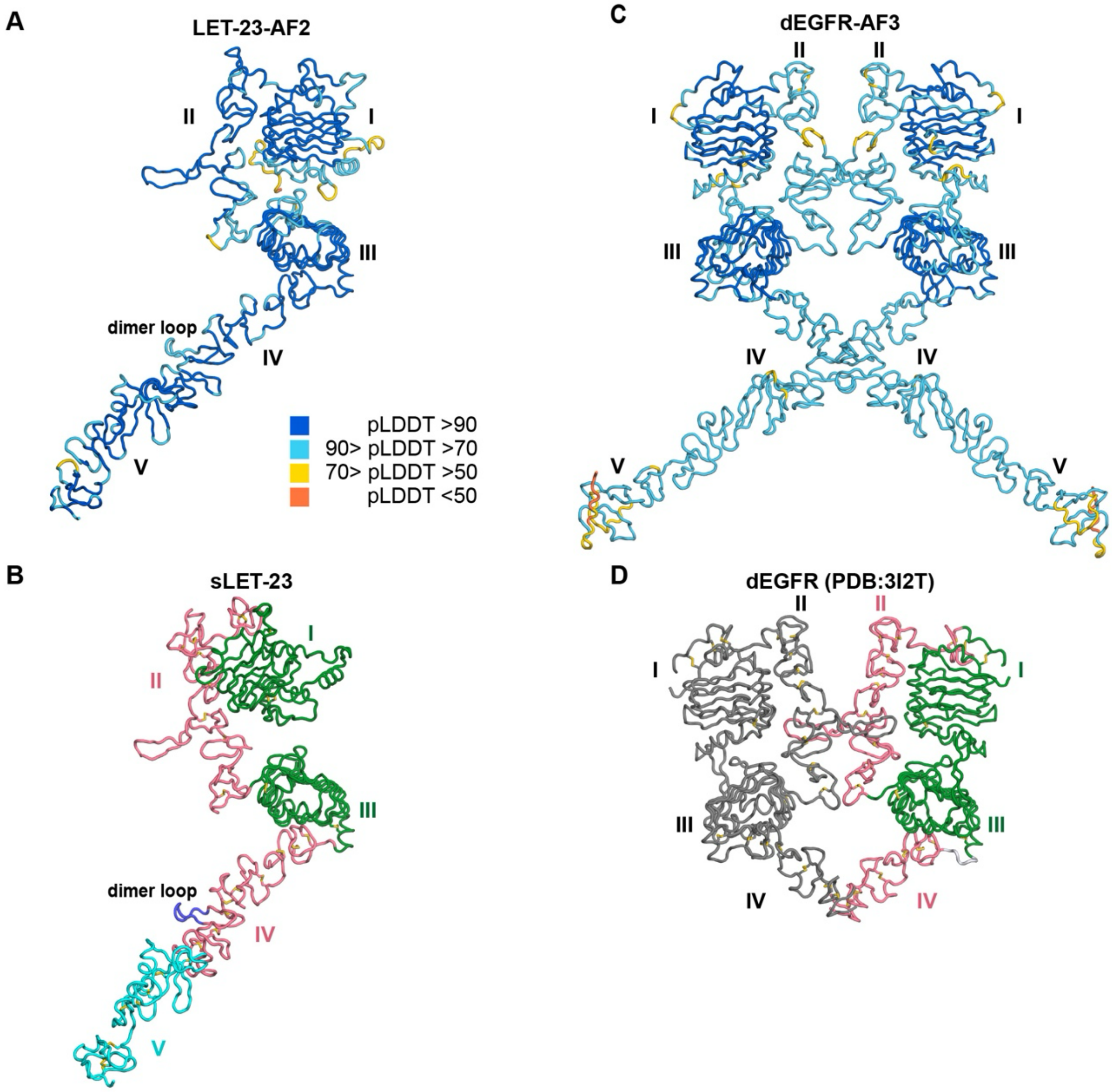

Fig. S7. AlphaFold Models
(*A*) An AlphaFold2 model (23) of a LET-23 monomer is shown in ribbon representation colored by model confidence, measured as the per-residue predicted local distance difference test (pLDDT) (15): blue for pLDDT> 90, pale blue for 90 < pLDDT > 70, yellow for < pLDDT >50, and orange for pLDDT < 50. The orientation is similar to that shown in Fig. 1*B*. The dimer loop structure is predicted with confidence. (*B*) A cartoon representation of one protomer from the final model of sLET-23 in the same orientation as (*A*) and using domain III to overlay this sLET-23 protomer on LET-23-AF2 shown in *A*. Domains are colored as in Fig. 1*A* and the domain IV dimer loop is in blue. (*C*) A ribbon representation of an AlphaFold3 model (24) for a dEGFR dimer, colored as in *A*. (*D*) A ribbon representation of the X-ray crystal structure of domains I-IV of dEGFR (PDB:3I2T) (27), colored as in Fig. 5*D*. Structures in *C* and *D* were overlaid using the domain II dimer arm.

Tables

Table S1. Cryo-EM data collection, refinement and validation statistics.

	sLET-23	LIN-3/sLET-23 From dataset 1	LIN-3/sLET-23^{inact} From dataset 2	sLET-23$^{\Delta loop}$	LIN-3/sLET-23$^{\Delta loop}$
EMDB code	EMDB-73275	EMDB-73276	EMDB-73277	EMDB-73278	EMDB-73279
PDB core	9YOR	9YOS	9YOT	9YOU	9YOV
**Data collection and processing**					
Microscope / Detector	FEI Titan Krios	FEI Titan Krios	FEI Titan Krios	FEI Titan Krios	Glacios
Magnification	105,000	105,000	105,000	105,000	45,000
Voltage (kV)	300	300	300	300	200
Electron exposure (e^-/ Å^2)	50	50	50	50	40
Pixel size (Å)	0.825	0.825	0.825	0. 825	0.868
Defocus range (μm)	-0.8 to -2.0	-0.8 to -2.0	-0.8 to -2.0	-0.8 to -2.0	-0.8 to -2.0
**Cryo-EM reconstruction**					
Symmetry imposed	C2	C2	C2	C2	C2
Initial particle images	2,900,000	1,400,000	1,600,000	3,900,000	709,000
Final particles images	298,605	285,565	267,599	155,789	148,822
Map resolution (Å)	2.90	2.37	2.81	4.02	3.36
FSC threshold	0.143	0.143	0.143	0.143	0.143
**Refinement**					
Initial model used (PDB code	9YOS	AlphaFold2 (15)	9YOS	9YOS	9YOS
Model resolution (Å)	2.90	2.40	2.81	4.20	3.36
Model composition					
Non-hydrogen atoms	12,180	13,294	13,074	12,044	13,176
Protein residues	1,494	1,636	1,602	1,480	1,624
Ligands	-	-	-	-	-
B factors (Å)					
Protein	198.3	135.1	190.7	476.2	150.7
Ligand	-	-	-	-	-
R.M.S. deviations					
Bond lengths (Å)	0.003	0.004	0.007	0.003	0.006
Bond angles (º)	0.681	0.693	0.772	0.686	0.746
Validation					
MolProbity score	2.07	2.20	2.09	2.03	2.00
Clashscore	9.73	9.23	9.78	11.75	11.3
Poor rotamers (%)	1.53	2.21	1.29	0	0.13
Ramachandran analysis					
Favored (%)	93.69	92.94	91.84	93.16	93.44
Allowed (%)	6.04	6.82	7.72	6.57	6.06
Disallowed (%)	0.27	0.25	0.44	0.27	0.5

Table S2. Details for homology-directed repair to introduce the domain IV mutants into the *let-23* locus.

Coding changes	Oligonucleotide sequence	Site(s) made	Site lost
L559A Q562A M565A	CGTTGCAGGTTTCACATTCCGGAGAACATCTTTCGCATTTAGCGCTTGTAG CGTTGCGAGCAAATCTAAATCAAAGCGGTATTTACTTTTTCAGACTACAC	HaeII	FspI
L559A N561A Q562A M565A	CGTTGCAGGTTTCACATTCCGGAGAACATCTTTCGCATTTAGCGCTTGTAG CAGCGCGAGCAAATCTAAATCAAAGCGGTATTTACTTTTTCAGACTACAC	HaeII	FspI
Δ594-602+GG	CTAGGTGAACTGGATTGTCTCACTTGCAGACATAAAACTCTCGGAGGAGAA TGTGTGCACGATTGTCCAGTTTCACACTTTCCGACGCAGAAAAATGTA	BseRI, ApaLI	-

Table S3. Details of the mutant alleles generated in the study.

Coding changes	Nickname	Sequence used	Alleles generated
L559A Q562A M565A	*let-23*(3A)	TTTCGCATTTCATTGATGTC	*sy2083*, *sy2084*
L559A N561A Q562A M565A	*let-23*(4A)	TTTCGCATTTCATTGATGTC	*sy2082*, *sy2088*
Δ594-602+GG	*let-23(Δloop)*	ACATAAAACTCTCTACAATT	*sy2089*, *sy2090*

Legends for SI Datasets

Dataset S1 (separate file): SE-AUC data for sLET-23$^{\Delta loop}$

Linearized SE-AUC data for three independent samples of sLET-23$^{\Delta loop}$ with and without LIN-3 (see Extended Methods for details).

Dataset S2 (separate file): Weakening LET-23 dIV interactions suppresses the *lin-3(n378)* Vul phenotype

The number of animals that were severely egg-laying-defective (Egl) two days after being picked as late-stage L4 larvae is given (# Egl), along with the total number of animals scored (# Total) and the resulting percentage Egl. The *let-23* genotype is indicated as follows: "*let-23(+)*" is the wild-type allele; "*let-23(3A)*" is *let-23(sy2083)* or *let-23(sy2084)*, which cause identical molecular changes (s*y2084* is indicated with an asterisk; "*let-23(4A)*" is *let-23(sy2082);* "*let-23(Δloop)*" is *let-23(sy2089)* or *let-23(sy2090)*, which case identical molecular changes at the *let-23* locus (*sy2090* is indicated with an asterisk). The *lin-3* genotype is indicated either as "*lin-3(+)*" (the wild-type allele) or "*lin-3(n378)*" (a partial loss-of-function allele). Data for Fisher's Exact Test are also listed.

Dataset S3 (separate file): Weakening LET-23 dIV interactions suppresses the *lin-2(n105)* Vul phenotype

The number of animals that were severely egg-laying-defective (Egl) two days after being picked as late-stage L4 larvae is given (# Egl), along with the total number of animals scored (# Total) and the resulting percentage Egl. The *let-23* genotype is indicated as follows: "*let-23(+)*" is the wild-type allele; "*let-23(3A)*" is *let-23(sy2083)*; "*let-23(4A)*" is *let-23(sy2082);* "*let-23(Δloop)*" is *let-23(sy2089)*. The *lin-2* genotype is indicated either as "*lin-2(+)*" (the wild-type allele) or "*lin-2(n105)*" (a partial loss-of-function allele). Data for Fisher's Exact Test are also listed.

Dataset S4 (separate file): Weakening LET-23 dIV interactions enhances delay in development to adulthood in *lin-15AB(n765)*

The percentage of animals (n=50) that were adults (had completed vulval morphogenesis) at the indicated number of hours following transfer of arrested L1 larvae to NGM plates with lawns of OP50 bacteria as a food source (see Extended Methods for details). Genotype is indicated as follows: "+" is for N2 (Bristol) wild type animals; "*let-23(3A)*" is *let-23(sy2083)*; "*let-23(4A)*" is *let-23(sy2082);* "*let-23(Δloop)*" is *let-23(sy2089)*. The *lin-15AB(n765)* is written as "n765" to save space.

Dataset S5 (separate file): Weakening LET-23 dIV interactions enhances penetrance of the SynMuv phenotype

Numbers of animals with no extra pseudovulvae ("blips"), with one extra pseudovulva, or with two or more extra pseudovulvae. Animals were grown from eggs or from developmentally arrested L1 larvae at the indicated temperatures (see Extended Methods for details). All animals are in the indicated synMuv double mutant background, with the *let-23* genotype indicated as follows: "*let-23(+)*" is the wild-type allele; "*let-23(3A)*" is *let-23(sy2083)*; "*let-23(4A)*" is *let-23(sy2082);* "*let-23(Δloop)*" is *let-23(sy2089)*.

SI References

1. S. Brenner, The genetics of *Caenorhabditis elegans*. *Genetics* **77**, 71–94 (1974).
2. E. L. Ferguson, H. R. Horvitz, Identification and characterization of 22 genes that affect the vulval cell lineages of the nematode *Caenorhabditis elegans*. *Genetics* **110**, 17–72 (1985).
3. H. R. Horvitz, J. E. Sulston, Isolation and genetic characterization of cell-lineage mutants of the nematode *Caenorhabditis elegans*. *Genetics* **96**, 435–454 (1980).
4. W. S. Katz *et al.*, A point mutation in the extracellular domain activates LET-23, the *Caenorhabditis elegans* epidermal growth factor receptor homolog. *Mol. Cell. Biol.* **16**, 529–537 (1996).
5. J. H. Thomas, C. J. Ceol, H. T. Schwartz, H. R. Horvitz, New genes that interact with *lin-35* Rb to negatively regulate the *let-60 ras* pathway in *Caenorhabditis elegans*. *Genetics* **164**, 135–151 (2003).
6. M. L. Edgley, D. L. Riddle, LG II balancer chromosomes in *Caenorhabditis elegans*: mT1(II;III) and the mIn1 set of dominantly and recessively marked inversions. *Mol. Genet. Genomics* **266**, 385–395 (2001).
7. K. Dejima *et al.*, An Aneuploidy-Free and Structurally Defined Balancer Chromosome Toolkit for *Caenorhabditis elegans*. *Cell Rep.* **22**, 232–241 (2018).
8. D. M. Freed, D. Alvarado, M. A. Lemmon, Ligand regulation of a constitutively dimeric EGF receptor. *Nat. Commun.* **6**, 7380 (2015).
9. J. Scholz, S. Suppmann, A new single-step protocol for rapid baculovirus-driven protein production in insect cells. *BMC Biotechnol.* **17**, 83 (2017).
10. D. E. Klein, S. E. Stayrook, F. Shi, K. Narayan, M. A. Lemmon, Structural basis for EGFR ligand sequestration by Argos. *Nature* **453**, 1271–1275 (2008).
11. D. N. Mastronarde, Automated electron microscope tomography using robust prediction of specimen movements. *J. Struct. Biol.* **152**, 36–51 (2005).
12. A. Punjani, J. L. Rubinstein, D. J. Fleet, M. A. Brubaker, cryoSPARC: algorithms for rapid unsupervised cryo-EM structure determination. *Nat. Methods* **14**, 290–296 (2017).
13. T. Bepler *et al.*, Positive-unlabeled convolutional neural networks for particle picking in cryo-electron micrographs. *Nat. Methods* **16**, 1153–1160 (2019).
14. R. Sanchez-Garcia *et al.*, DeepEMhancer: a deep learning solution for cryo-EM volume post-processing. *Commun. Biol.* **4**, 874 (2021).
15. J. Jumper *et al.*, Highly accurate protein structure prediction with AlphaFold. *Nature* **596**, 583–589 (2021).
16. E. C. Meng *et al.*, UCSF ChimeraX: Tools for structure building and analysis. *Protein science : a publication of the Protein Society* **32**, e4792 (2023).
17. P. Emsley, B. Lohkamp, W. G. Scott, K. Cowtan, Features and development of Coot. *Acta Crystallogr. D Biol. Crystallogr.* **66**, 486–501 (2010).
18. A. Casañal, B. Lohkamp, P. Emsley, Current developments in Coot for macromolecular model building of Electron Cryo-microscopy and Crystallographic Data. *Protein Sci.* **29**, 1069–1078 (2020).
19. P. D. Adams *et al.*, PHENIX: a comprehensive Python-based system for macromolecular structure solution. *Acta Crystallogr. D Biol. Crystallogr.* **66**, 213–221 (2010).
20. Anonymous (The PyMOL Molecular Graphics System, Version 3.1.0, Schrödinger, LLC.
21. E. Krissinel, K. Henrick, Inference of macromolecular assemblies from crystalline state. *J. Mol. Biol.* **372**, 774–797 (2007).
22. S. Hayward, H. J. Berendsen, Systematic analysis of domain motions in proteins from conformational change: new results on citrate synthase and T4 lysozyme. *Proteins* **30**, 144–154 (1998).
23. M. Mirdita *et al.*, ColabFold: making protein folding accessible to all. *Nat. Methods* **19**, 679–682 (2022).
24. J. Abramson *et al.*, Accurate structure prediction of biomolecular interactions with AlphaFold 3. *Nature* **630**, 493–500 (2024).
25. J. A. Arribere *et al.*, Efficient marker-free recovery of custom genetic modifications with CRISPR/Cas9 in *Caenorhabditis elegans*. *Genetics* **198**, 837–846 (2014).

26. P. M. Sharp, K. R. Bradnam, "Appendix 3 Codon Usage in *C. elegans*" in *C. elegans* II 2nd edition, D.L.Riddle, T. Blumenthal, B.J. Meyer, *et al*. Eds. (Cold Spring Harbor Laboratory Press, 1997).
27. D. Alvarado, D. E. Klein, M. A. Lemmon, ErbB2 resembles an autoinhibited invertebrate epidermal growth factor receptor. *Nature* **461**, 287–291 (2009).
